# Statistical inference and data analysis for inverted Kumaraswamy distribution based on maximum ranked set sampling with unequal samples

**DOI:** 10.1038/s41598-024-74468-4

**Published:** 2024-10-26

**Authors:** Amal S. Hassan, Samah A. Atia

**Affiliations:** Faculty of Graduate Studies for Statistical Research, Cairo University, Giza, 12613 Egypt

**Keywords:** Inverted Kumaraswamy distribution, Maximum ranked set sampling with unequal size, Maximum likelihood estimation, Bayes estimation, Markov Chain Monte Carlo, Ecology, Mathematics and computing

## Abstract

A very useful modification to ranked set sampling (RSS) that allows a larger set size without significantly increasing ranking errors is the maximum ranked set sampling with unequal samples (MRSSU) approach. This article covers the parameter estimation of the inverted Kumaraswamy distribution using MRSSU and RSS designs. The maximum likelihood and Bayesian estimation techniques are considered. The regarded Bayesian estimation technique is determined in the case of non-informative and informative priors represented by Jeffreys and gamma priors, respectively. Squared error and minimum expected are the two loss functions that are employed. We presented a simulation study to evaluate the performance of the recommended estimations using root mean squared error and relative bias. The Bayes point estimates were computed using the Metropolis–Hastings algorithm. Additional conclusions have been made based on actual geological data regarding the intervals between Kiama Blowhole’s 64 consecutive eruptions. Based on the same number of measured units, the results of simulation and real data analysis showed that MRSSU estimators performed much better than their RSS counterparts.

## Introduction

In certain studies, cost-effective sampling is a major issue, especially if measuring the relevant trait is costly, painful, or time-consuming. Utilizing the ranked set sampling (RSS) technique, which ranks data according to the variable of interest but makes measuring units in a population expensive, is a successful approach to gathering data. McIntyre^1^ introduced RSS methodology as a substitute for the widely utilized simple random sample (SRS) technique. The RSS method has applications in different areas such as industrial (Taylan et al.^2^), agricultural (Husby et al.^3^), environmental and ecological studies (Mode et al.^4^, and Ozturk et al.^5^), agriculture (Bocci et al.^6^), biology (Halls and Dell^7^), engineering applications (Nadarajah and Kotz^8^) and geology (Lawless^9^). The theoretical features of this technique were investigated by Takahasi and Wakimoto^10^ and Dell and Clutter^11^ under the assumptions of imperfect and perfect judgment ranking, respectively. It was demonstrated by Dell and Clutter^11^ that regardless of whether judgment ranking is perfect, the variation for the RSS mean is never more than the variance for the SRS mean. For further work on parametric approaches for RSS (see, for example, Lam et al.^12^, and Stokes^13^).

RSS is a more effective sampling technique than the SRS technique for estimating the population mean. When the number of observations is equal, the variance of RSS is consistently lower than that of SRS. The fundamental concept of sample selection under RSS is as follows: *m*^2^ randomly selected units are taken from the parent population, and *m*^2^ randomly selected units are customized into *m* sets, each of size *m*. The smallest to largest datasets in each dataset are sorted, which implies:

$$\left( {\begin{array}{*{20}l} {\underline{{\boxed{X_{{(1)1}} }}} } \hfill & {X_{{(1)2}}^{{}} } \hfill & \ldots \hfill & {X_{{(1)m}}^{{}} } \hfill \\ {X_{{(2)1}}^{{}} } \hfill & {\underline{{\boxed{X_{{(2)2}}^{{}} }}} } \hfill & \ldots \hfill & {X_{{(2)m}}^{{}} } \hfill \\ \vdots \hfill & \vdots \hfill & \ddots \hfill & \vdots \hfill \\ {X_{{(m)1}}^{{}} } \hfill & {X_{{(m)2}}^{{}} } \hfill & \ldots \hfill & {\underline{{\boxed{X_{{(m)m}}^{{}} }}} } \hfill \\ \end{array} } \right),$$

where the obtained RSS is (*X*_(1)1_, *X*_(2)2_,…, *X*_(*m*)*m*_) with one-cycle. If this method is repeated *n* times, then *X*(*i*)*ij* denotes the ranked units of the *ith* ordered statistics from the *ith* set of size *m* in the *jth* cycle of size *n*, where (*i* = 1, 2, …, *m*, *j* = 1, 2, …, *n*), and the sample size is $n^\circ = mn.$ For simpler notation, we can write *X*_*ij*_ instead of *X*_(*i)ij*_.

Since RSS’s release, several modifications have been proposed to improve the effectiveness of RSS-based inference. The maximum RSS with unequal samples (MRSSU), which was introduced by Biradar and Santosha^14^, is one of the most significant modifications. This approach is an enormously useful RSS adaptation. It permits growth in set size without creating an excessive number of ranking errors and helps to optimize the use of available resources while maintaining or even enhancing the quality of statistical inferences. It arises in environmental research (Jiang and Gui^15^), information theory (Eskandarzadeh et al.^16^ and Eskandarzadeh et al.^17^), biophysics (Mohie El-Din et al.^18^), and reliability engineering (Salehi and Ahmadi^19^ and Hassan et al.^20^). The MRSSU scenario is conducted with ${{m(m + 1)} \mathord{\left/ {\vphantom {{m(m + 1)} 2}} \right. \kern-0pt} 2}$ units in the sampling procedure. It is assumed that ${{m(m + 1)} \mathord{\left/ {\vphantom {{m(m + 1)} 2}} \right. \kern-0pt} 2}$ units are divided into *m* groups. For the *ith* (*i* = 1, 2, …, *m*) group with size *i*, the largest observation is selected from each group in the ranking process, and the MRSSU sample is then obtained as (*Y*_(1)1_, *Y*_(2)2_,…, *Y*_(*m*)*m*_). Similarly, the MRSSU procedure is described as follows:

$$\left( {\begin{array}{*{20}l} {\underline{{\boxed{Y_{{(1)1}} }}} } \hfill & {} \hfill & {} \hfill & {} \hfill \\ {Y_{{(2)1}}^{{}} } \hfill & {\underline{{\boxed{Y_{{(2)2}}^{{}} }}} } \hfill & {} \hfill & {} \hfill \\ \vdots \hfill & \vdots \hfill & \ddots \hfill & {} \hfill \\ {Y_{{(m)1}}^{{}} } \hfill & {Y_{{(m)2}}^{{}} } \hfill & \ldots \hfill & {\underline{{\boxed{Y_{{(m)m}}^{{}} }}} } \hfill \\ \end{array} } \right).$$

To get $n^\circ = mn$ sample units, the cycle can be repeated *n* times, where *Y*_(*i*)*ij*_ is the *ith* recorded units in *jth* cycle (*i* = 1, 2, …, *m*, *j* = 1, 2, …, *n*). For simpler notation, we can write *Y*_*ij*_ instead of *Y*_(*i)ij*_.

Many authors have examined estimation problems in various distribution scenarios. For example, Sadek et al.^21^ used the asymmetric loss function to obtain the Bayesian estimate (BE) of the exponential distribution based on SRS and RSS. Estimation for the unknown parameters of the Weibull distribution under different samples has been investigated by Helu et al.^22^. With RSS, Hassan^23^ was able to obtain the BEs and maximum likelihood estimates (MLEs) for the exponentiated exponential distribution. Based on SRS and RSS, Sadek and Alharbi^24^ employed a loss function to get the BE of the exponential and Weibull distributions, respectively. Biradar and Santosha^14^ discussed the estimation of exponential distribution parameter using MRSSU and showed that the MRSSU is better than those of the estimator based on SRS. Biradar and Shivanna^25^ examined the BEs of the Weibull distribution parameters under RSS and MRSS. Using RSS and MRSSU, BE of the exponential and Rayleigh distributions’ parameters was investigated by Eskandarzadeh et al.^16^. Under the RSS approach, Bantan et al.^26^ produced estimators of the Zubair Lomax distribution parameter. Using advanced sampling techniques, Almarashi et al.^27^ investigated the reliability estimate of the Topp-Leone distribution. Using various techniques for RSS, Jiang and Gui^15^ and Nagy et al.^28^ discussed, respectively, the estimate of Kumaraswamy distribution and inverted Kumaraswamy distribution parameters. The estimation of exponentiated exponential distribution under RSS, partial RSS, and neoteric RSS was presented by Hassan et al.^29^. When samples are unequal in size, Chaudhary and Gupta^30^ defined the general weighted extropy of the lowest RSS and MRSS. Wang et al.^31^ presented the estimation for inverse Gaussian distribution using MRSSU. The reader may refer to these sources [Hassan et al.^32^, Özdemir et al.^33^, Al-Omari et al.^34^, Al-Omari et al.^35^, Al-Omari et al.^36^, Hassan et al.^37^, Alsadat et al.^38,39^ and Liu et al.^40^] for additional research.

Inverted distributions have been shown to be efficient in the fields of econometrics, engineering science, pharmaceutical applications, biology, geology, and life testing challenges. The inverted Kumaraswamy distribution (IKmD), presented by Abd Al-Fattah et al.^41^, is one of the most useful models. The IKmD was created as the inverse form of the Kumaraswamy distribution. For application of the IKmD in different fields, the reader can refer to Rashad et al.^42^ and Nagy et al.^28^. The cumulative distribution function (CDF), probability density function (PDF) and hazard rate function (HRF) for the random variable *X* are defined, respectively, by

1 $$F(x;\gamma ,\tau ) = \left[ {{1} - {\kern 1pt} {\kern 1pt} {(1 + }x{)}{\kern 1pt}^{ - \gamma } {\kern 1pt} } \right]^{\tau } ,\quad \quad \quad x > 0,$$

2 $$f(x;\gamma ,\tau ) = \gamma \,\tau \left( {1 + x} \right)^{ - \gamma - 1} \left[ {1 - {\kern 1pt} {\kern 1pt} (1 + x){\kern 1pt}^{ - \gamma } {\kern 1pt} } \right]^{\tau - 1} ,\quad \quad \quad x > 0,$$

and

$$h(x;\gamma ,\tau ) = \frac{{\gamma \,\tau \left( {1 + x} \right)^{ - \gamma - 1} \left[ {1 - {\kern 1pt} {\kern 1pt} (1 + x){\kern 1pt}^{ - \gamma } {\kern 1pt} } \right]^{\tau - 1} }}{{1 - \left[ {{1} - {\kern 1pt} {\kern 1pt} {(1 + }x{)}{\kern 1pt}^{ - \gamma } {\kern 1pt} } \right]^{\tau } }},\quad \quad \quad x > 0,$$

where $\gamma > 0,$ and $\tau > 0,$ are the shape parameters. The IKmD has a lengthy right tail, when measured against other distributions. As a result, it will have an impact on long-term reliability estimates, resulting in optimistic predictions of uncommon occurrences that will occur in the right tail of the distribution relative to other distributions (see Abd Al-Fattah et al.^41^).

**Fig. 1:**
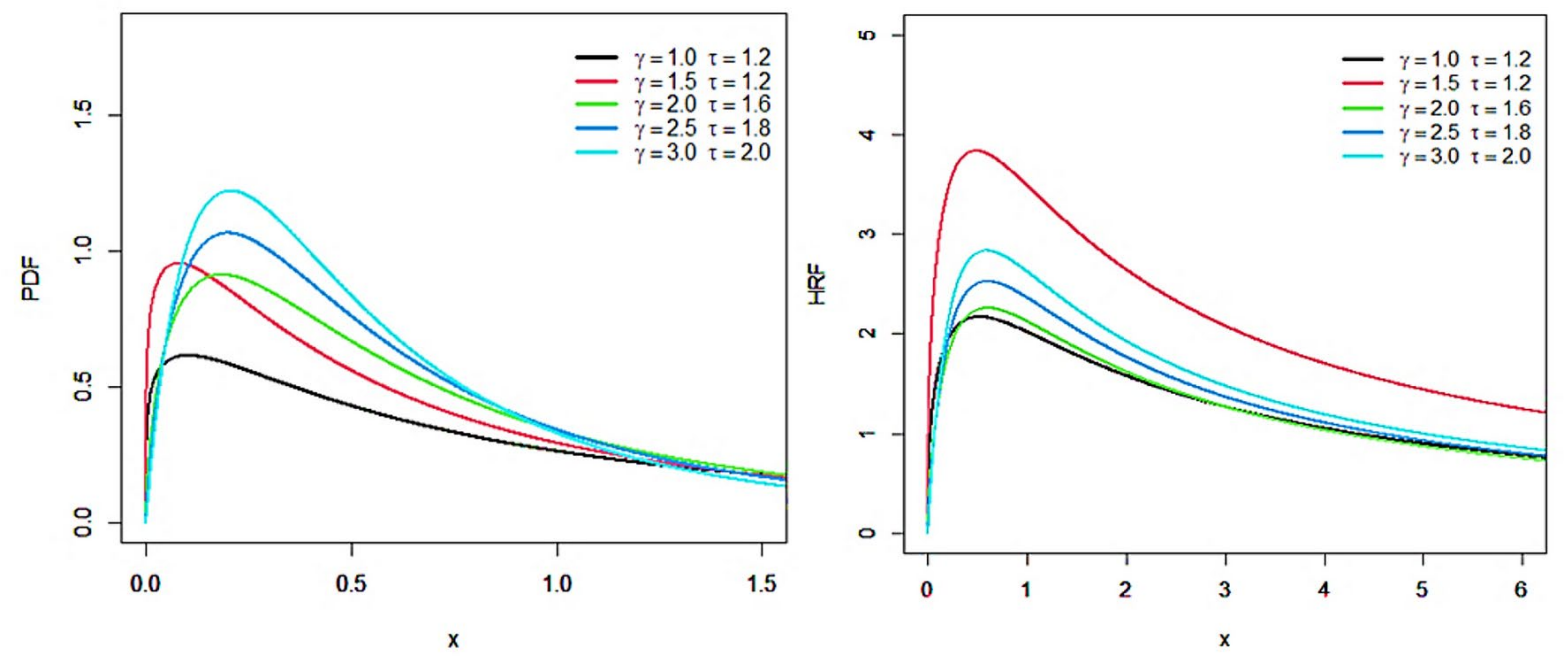
Plots of the PDF and HRF of the IKmD.

The PDF and HRF charts are shown in Fig. 1. This figure illustrates how the PDF and HRF of the IKmD take different shapes. There are several helpful forms for the PDF in Fig. 1. The graphical representation in Fig. 1 shows that the HRF of the IKmD distribution can have a variety of forms, including growing, decreasing, and upside-down.

In light of the importance of MRSSU design in reducing observation ranking error, enhancing statistical inference, and various applications of the IKmD, this work explores the estimate of IKmD parameters through the use of RSS and MRSSU techniques. Specifically, we employ both maximum likelihood and Bayesian estimation frameworks. For Bayesian inference, we consider informative priors (IFP; gamma prior) and non-informative priors (NIFP; Jeffreys prior), coupled with squared error loss function (SELoF) and minimum expected loss function (MELoF). To sample from the posterior distribution, we implement the Metropolis–Hastings (MH) algorithm. The performance of these estimators is evaluated across various sample sizes using Markov Chain Monte Carlo (MCMC) technique and metrics such as root mean squared error (RMSE) and relative absolute bias (RAB). Finally, we illustrate the proposed methodology through a real-world application.

The rest of the paper is organized as follows: Sect. “Estimation methods based on RSS” provides the estimation of parameters using RSS. Section “Estimation methods based on MRSSU” provides the estimation of parameters using MRSSU. Sections “Simulation result” and “Application for real data” discuss, respectively, simulation studies and their applications to actual circumstances, as well as a comparison of MRSSU estimators to RSS equivalents. Finally, concluding remarks are handled in Sect. “Conclusions”.

## Estimation methods based on RSS

This section examines the MLEs and BEs for estimating the IKmD parameters based on RSS.

### Maximum likelihood estimators

Let {*X*_*ij*_*, i* = 1,2,…,*m*, *j* = 1,2,…,*n*} denote the RSS of size $n^\circ = mn$ from the IKmD with parameters $\gamma ,\tau > 0$, where *m* is the set size and *n* is the cycle count. According to Takahasi and Wakimoto^10^, the PDF of *X*_*ij*_, under the assumption of perfect ranking, is exactly the PDF of *i*th order statistics given by:

3 $$f(x_{ij} ) = \,\frac{m!}{{(i - 1)!(m - i)! \, }}\;{\kern 1pt} f( \, x_{(ij)} ;\gamma ,\tau )\left[ {F(x_{(ij)} ;\gamma ,\tau )} \right]^{i - 1} \, [1 - F(x_{(ij)} ;\gamma ,\tau )]^{m - i} \;,\quad x,\;\gamma ,\;\tau > 0.$$

Then the likelihood function is provided by using CDF (1) and PDF (2) in Eq. (3) as below:

4 $$L_{1} (\left. {\underline{x} } \right|\gamma ,\tau ) = \,\,\tau^{n^\circ } {\kern 1pt} \gamma^{n^\circ } \prod\limits_{j = 1}^{n} {\prod\limits_{i = 1}^{m} {i\left( {\begin{array}{*{20}c} m \\ i \\ \end{array} } \right)\;{\kern 1pt} \left[ {1 - {\kern 1pt} (1 + x_{ij} ){\kern 1pt}^{ - \gamma } {\kern 1pt} } \right]^{i\tau - 1} \left( {1 + x_{ij} } \right)^{ - \gamma - 1} \left[ {1 - \left[ {1 - {\kern 1pt} {\kern 1pt} (1 + x_{ij} ){\kern 1pt}^{ - \gamma } {\kern 1pt} } \right]^{\tau } } \right]^{m - i} \,} } .$$

The log-likelihood function for Eq. 4 is:

5 $$\begin{gathered} l_{1} (\left. {\underline{x} } \right|\gamma ,\tau )\, \propto \,n^\circ \,\ln \,\tau_{{}}^{{}} + n^\circ \,\ln \,\gamma_{{}}^{{}} + \sum\limits_{j = 1}^{n} {\sum\limits_{i = 1}^{m} {(i\tau - 1)\ln \left[ {\Phi (x_{ij} ,\gamma )} \right]\,} } - (\gamma + 1)\sum\limits_{j = 1}^{n} {\sum\limits_{i = 1}^{m} {\ln } \left( {1 + x_{ij} } \right)} \hfill \\ \quad \quad \quad + \sum\limits_{j = 1}^{n} {\sum\limits_{i = 1}^{m} {(m - i)\ln \left[ {1 - \left[ {\Phi (x_{ij} ,\gamma ){\kern 1pt} } \right]^{\tau } } \right],\,} } \hfill \\ \end{gathered}$$

where $\Phi (x_{ij} ,\gamma ) = 1 - {\kern 1pt} {\kern 1pt} (1 + x_{ij} ){\kern 1pt}^{ - \gamma } .$ The partial derivatives of Eq. 5 with respect to $\gamma ,$ and $\tau$ are as follows:

6 $$\begin{gathered} \frac{{\partial \;l_{1} (\left. {\underline{x} } \right|\gamma ,\tau )}}{\partial \gamma }{\kern 1pt} = \frac{n^\circ }{\gamma } - \sum\limits_{j = 1}^{n} {\sum\limits_{i = 1}^{m} {\ln } \left( {1 + x_{ij} } \right)} \; + \sum\limits_{j = 1}^{n} {\sum\limits_{i = 1}^{m} {\frac{{(i\tau - 1)\;\ln (1 + x_{ij} )\;\;\;}}{{(1 + x_{ij} )^{\gamma } - 1}}\,} } \hfill \\ \, - \sum\limits_{j = 1}^{n} {\sum\limits_{i = 1}^{m} {\frac{{\tau (m - i)\;(1 + x_{ij} ){\kern 1pt}^{ - \gamma } \;\;\ln \left( {1 + x_{ij} } \right)\left[ {\Phi (x_{ij} ,\gamma ){\kern 1pt} } \right]^{\tau - 1} }}{{1 - \left[ {\Phi (x_{ij} ,\gamma ){\kern 1pt} } \right]^{\tau } }}\,} } , \hfill \\ \end{gathered}$$

and,

7 $$\frac{{\partial \;l_{1} (\left. {\underline{x} } \right|\gamma ,\tau )}}{\partial \tau }{\kern 1pt} {\kern 1pt} = \,\frac{n^\circ }{\tau } + \sum\limits_{j = 1}^{n} {\sum\limits_{i = 1}^{m} {i\ln \left[ {\Phi (x_{ij} ,\gamma )} \right] - \,} } \sum\limits_{j = 1}^{n} {\sum\limits_{i = 1}^{m} {\frac{{(m - i)\ln \left( {\Phi (x_{ij} ,\gamma )} \right)}}{{\left[ {\Phi (x_{ij} ,\gamma ){\kern 1pt} } \right]^{ - \tau } - 1}}} } .$$

The principle of the ML method is to estimate a parameter $\gamma ,$ and $\tau$ by finding the value of $\hat{\gamma }_{{1}} ,$ and $\hat{\tau }_{1}$ by solving Eqs. 6 and 7, which maximize the log-likelihood:

$$\left. {\frac{{\partial \;l_{1} (\left. {\underline{x} } \right|\gamma ,\tau )}}{\partial \gamma }} \right|_{{\gamma = \hat{\gamma }_{1} }} {\kern 1pt} = 0, \;\;\text{and}\;\; \left. {\frac{{\partial \;l_{1} (\left. {\underline{x} } \right|\gamma ,\tau )}}{\partial \tau }} \right|_{{\tau = \hat{\tau }_{1} }} {\kern 1pt} = 0.$$

It is difficult to obtain a closed-form solution for these equations, so numerical procedure is required to solve them by the R 4.3.0 program using package “bbmle”.

### Bayesian estimators

The Bayesian method is used to obtain the estimators of the IKmD parameters based on RSS. The BEs are derived under IFP and NIFP, represented by gamma and uniform priors, respectively. The primary challenge in executing the Bayesian process is acquiring the posterior distribution. The MH algorithm is one of the most well-known subclasses of the MCMC method in Bayesian literature for simulating deviations from the posterior density and generating accurate approximations, see Ravenzwaaij et al.^43^.

The BEs in the case of IFP will be obtained assuming the shape parameters $\gamma$ and $\tau$ have a gamma distribution with parameters *a*, *b, c*, and *d,* respectively. Assuming independence of parameters, the joint prior distribution of parameters $\gamma ,$ and $\tau$ is given by:

8 $$g_{1,2} (\gamma ,\tau ) \propto \,\,\,\gamma^{a - 1} \,\tau^{c - 1} \,e^{ - d\tau - b\gamma } ;\,\,\,\,\,\,a,b,c,d > 0.$$

Using binomial expansion, the last term of Eq. 4 can be written as:

$$\left[ {1 - {\kern 1pt} (1 + x_{ij} ){\kern 1pt}^{ - \gamma } {\kern 1pt} } \right]^{i\tau - 1} = \sum\limits_{{l_{i} = 0}}^{\infty } {\left( {\begin{array}{*{20}c} {i\tau - 1} \\ {l_{i} } \\ \end{array} } \right)\left( { - 1} \right)^{{l_{i} }} {\kern 1pt} \left( {1 + x_{i} } \right)} {\kern 1pt}^{{ - \gamma l_{i} }} .$$

Then the Eq. 4 for likelihood function is given by:

9 $$L_{1} (\left. {\underline{x} } \right|\gamma ,\tau ) = \,\tau^{n^\circ } \,\gamma^{n^\circ } \;{\kern 1pt} \left[ {\sum\limits_{{l_{1}^{1} = 0}}^{0} {\;\sum\limits_{{l_{2}^{1} = 0}}^{1} {...\sum\limits_{{l_{m}^{1} = 0}}^{m - 1} {} } } } \right]\left[ {\sum\limits_{{l_{1}^{2} = 0}}^{0} {\;\sum\limits_{{l_{2}^{2} = 0}}^{1} {...\sum\limits_{{l_{m}^{2} = 0}}^{m - 1} {} } } } \right]\;...\;\left[ {\sum\limits_{{l_{1}^{n} = 0}}^{0} {\;\sum\limits_{{l_{2}^{n} = 0}}^{1} {...\sum\limits_{{l_{m}^{n} = 0}}^{m - 1} {} } } } \right]\left[ {\prod\limits_{j = 1}^{n} {\prod\limits_{i = 1}^{m} {V_{{l_{i}^{j} }} } } } \right],$$

where $$V_{{l_{i}^{j} }} = \frac{{c_{{l_{i} }} (i)\left[ {1 - \left[ {\Phi (x_{ij} ,\gamma ){\kern 1pt} } \right]^{\tau } } \right]^{m - i} }}{{\left( {1 + x_{ij} } \right)^{{\gamma l_{i} + \gamma + 1}} }},$$ and $$c_{{l_{i} }} (i) = i\left( {\begin{array}{*{20}c} m \\ i \\ \end{array} } \right)\left( {\begin{array}{*{20}c} {i\tau - 1} \\ {l_{i} } \\ \end{array} } \right)\left( { - 1} \right)^{{l_{i} }} .$$

Based on Eqs. 8 and 9 for the joint prior distribution of parameters and the likelihood function, respectively, the joint posterior density of $\gamma ,$ and $\tau$ given the data $\underline{x}$ can be written as follows:

10 $$\pi_{1} (\gamma ,\tau \left| {\underline{x} } \right.) = \frac{{L_{1} (\left. {\underline{x} } \right|\gamma ,\tau )\;\;g_{1,2} (\gamma ,\tau )}}{{\int\limits_{\gamma } {\int\limits_{\tau } {L_{1} (\left. {\underline{x} } \right|\gamma ,\tau )\;g_{1,2} (\gamma ,\tau )\;d\tau \;d\gamma } } }}.$$

From Eqs. 8, 9, and 10, the joint posterior for $\gamma ,$ and $\tau$ can be written as follows:

$$\pi_{1} (\gamma ,\tau \left| {\underline{x} } \right.) \propto \tau_{{}}^{n^\circ + c - 1} \;\,\gamma_{{}}^{n^\circ + a - 1} \;e^{ - b\gamma - d\tau } \left[ {\sum\limits_{{l_{1}^{1} = 0}}^{0} {\;\sum\limits_{{l_{2}^{1} = 0}}^{1} {...\sum\limits_{{l_{m}^{1} = 0}}^{m - 1} {} } } } \right]\left[ {\sum\limits_{{l_{1}^{2} = 0}}^{0} {\;\sum\limits_{{l_{2}^{2} = 0}}^{1} {...\sum\limits_{{l_{m}^{2} = 0}}^{m - 1} {} } } } \right]\;...\;\left[ {\sum\limits_{{l_{1}^{n} = 0}}^{0} {\;\sum\limits_{{l_{2}^{n} = 0}}^{1} {...\sum\limits_{{l_{m}^{n} = 0}}^{m - 1} {} } } } \right]\left[ {\prod\limits_{j = 1}^{n} {\prod\limits_{i = 1}^{m} {V_{{l_{i}^{j} }} } } } \right].$$

Hence, the conditional posterior densities for $\gamma ,$ and $\tau$ take the following shapes

$$h_{1} (\left. \gamma \right|\tau,\underline{x} ) = \;K_{1}^{ - 1} \;\gamma^{n^\circ + a - 1} e^{ - b\gamma } \int\limits_{{0}}^{\infty } {\tau^{n^\circ + c - 1} e^{ - d\tau } \left[ {\sum\limits_{{l_{1}^{1} = 0}}^{0} {\;\sum\limits_{{l_{2}^{1} = 0}}^{1} {...\sum\limits_{{l_{m}^{1} = 0}}^{m - 1} {} } } } \right]\left[ {\sum\limits_{{l_{1}^{2} = 0}}^{0} {\;\sum\limits_{{l_{2}^{2} = 0}}^{1} {...\sum\limits_{{l_{m}^{2} = 0}}^{m - 1} {} } } } \right]\;...\;\left[ {\sum\limits_{{l_{1}^{n} = 0}}^{0} {\;\sum\limits_{{l_{2}^{n} = 0}}^{1} {...\sum\limits_{{l_{m}^{n} = 0}}^{m - 1} {} } } } \right]\left[ {\prod\limits_{j = 1}^{n} {\prod\limits_{i = 1}^{m} {V_{{l_{i}^{j} }} } } } \right]\;d\tau } ,$$

and,

$$h_{2} (\left. \tau \right|\gamma,\underline{x} ) = \;K_{1}^{ - 1} \tau^{n^\circ + c - 1} e^{ - d\tau } \;\int\limits_{{0}}^{\infty } {\gamma^{n^\circ + a - 1} e^{ - b\gamma } \left[ {\sum\limits_{{l_{1}^{1} = 0}}^{0} {\;\sum\limits_{{l_{2}^{1} = 0}}^{1} {...\sum\limits_{{l_{m}^{1} = 0}}^{m - 1} {} } } } \right]\left[ {\sum\limits_{{l_{1}^{2} = 0}}^{0} {\;\sum\limits_{{l_{2}^{2} = 0}}^{1} {...\sum\limits_{{l_{m}^{2} = 0}}^{m - 1} {} } } } \right]\;...\;\left[ {\sum\limits_{{l_{1}^{n} = 0}}^{0} {\;\sum\limits_{{l_{2}^{n} = 0}}^{1} {...\sum\limits_{{l_{m}^{n} = 0}}^{m - 1} {} } } } \right]\left[ {\prod\limits_{j = 1}^{n} {\prod\limits_{i = 1}^{m} {V_{{l_{i}^{j} }} } } } \right]\;d\gamma } ,$$

where,

$$\begin{aligned} K_{1} & = \int\limits_{0}^{\infty } {\int\limits_{0}^{\infty } {\tau ^{{n^\circ + c - 1}} \;\gamma ^{{n^\circ + a - 1}} \;e^{{ - b\gamma - d\tau }} \left[ {\sum\limits_{{l_{1}^{1} = 0}}^{0} {\;\sum\limits_{{l_{2}^{1} = 0}}^{1} { \ldots \sum\limits_{{l_{m}^{1} = 0}}^{{m - 1}} {} } } } \right]\left[ {\sum\limits_{{l_{1}^{2} = 0}}^{0} {\;\sum\limits_{{l_{2}^{2} = 0}}^{1} { \ldots \sum\limits_{{l_{m}^{2} = 0}}^{{m - 1}} {} } } } \right]} } \\ & \quad \ldots \;\left[ {\sum\limits_{{l_{1}^{n} = 0}}^{0} {\;\sum\limits_{{l_{2}^{n} = 0}}^{1} { \ldots \sum\limits_{{l_{m}^{n} = 0}}^{{m - 1}} {} } } } \right]\left[ {\prod\limits_{{j = 1}}^{n} {\prod\limits_{{i = 1}}^{m} {V_{{l_{i}^{j} }} } } } \right]\;d\tau d\gamma \\ \end{aligned}$$

Since the distributions of these marginal posterior distributions are unknown, we can deal with them using the MH approach.

i. Bayesian estimators under SELoF

One of the most practical symmetric loss functions is thought to be SELoF, which is defined as follows:

$$L_{SELoF} (\beta ,\tilde{\beta }) = (\tilde{\beta } - \beta )_{{}}^{2} .$$

The BE of $\beta =(\gamma, \tau)$ under SELoF is defined by:

11 $$\tilde{\beta }_{SELoF} = E(\beta \left| {\underline{x} } \right.) = \int\limits_{\beta }^{{}} {\beta \;\;w(\beta \left| {\underline{x} } \right.)} \;d\beta ,\;$$

where $\tilde{\beta }_{SELoF}$ is BE for $\beta$ under SELoF. Regarding Eq. 11, the BE of $\gamma$ under SELoF, denoted by $\tilde{\gamma }_{SELoF} ,$ can be obtained as a posterior mean as follows:

12 $$\tilde{\gamma }_{SELoF} = \frac{{\int\limits_{0}^{\infty } {\int\limits_{{0}}^{\infty } {\tau^{n^\circ + c - 1} \;\gamma^{n^\circ + a} e^{ - (b\gamma + d\tau )} \left[ {\sum\limits_{{l_{1}^{1} = 0}}^{0} {\;\sum\limits_{{l_{2}^{1} = 0}}^{1} {...\sum\limits_{{l_{m}^{1} = 0}}^{m - 1} {} } } } \right]\left[ {\sum\limits_{{l_{1}^{2} = 0}}^{0} {\;\sum\limits_{{l_{2}^{2} = 0}}^{1} {...\sum\limits_{{l_{m}^{2} = 0}}^{m - 1} {} } } } \right]\;...\;\left[ {\sum\limits_{{l_{1}^{n} = 0}}^{0} {\;\sum\limits_{{l_{2}^{n} = 0}}^{1} {...\sum\limits_{{l_{m}^{n} = 0}}^{m - 1} {} } } } \right]\left[ {\prod\limits_{j = 1}^{n} {\prod\limits_{i = 1}^{m} {V_{{l_{i}^{j} }} } } } \right]\;d\tau d\gamma } } }}{{\;\;\int\limits_{0}^{\infty } {\int\limits_{{0}}^{\infty } {\tau^{n^\circ + c - 1} \,\gamma^{n^\circ + a - 1} e^{ - (b\gamma + d\tau )} \left[ {\sum\limits_{{l_{1}^{1} = 0}}^{0} {\;\sum\limits_{{l_{2}^{1} = 0}}^{1} {...\sum\limits_{{l_{m}^{1} = 0}}^{m - 1} {} } } } \right]\left[ {\sum\limits_{{l_{1}^{2} = 0}}^{0} {\;\sum\limits_{{l_{2}^{2} = 0}}^{1} {...\sum\limits_{{l_{m}^{2} = 0}}^{m - 1} {} } } } \right]\;...\;\left[ {\sum\limits_{{l_{1}^{n} = 0}}^{0} {\;\sum\limits_{{l_{2}^{n} = 0}}^{1} {...\sum\limits_{{l_{m}^{n} = 0}}^{m - 1} {} } } } \right]\left[ {\prod\limits_{j = 1}^{n} {\prod\limits_{i = 1}^{m} {V_{{l_{i}^{j} }} } } } \right]\;d\tau d\gamma } } }}.$$

Similarly, the BE of $\tau$ under the SELoF, say $\tilde{\tau }_{SELoF} ,$ is given by:

13 $$\tilde{\tau }_{SELoF} = \frac{{\int\limits_{0}^{\infty } {\int\limits_{0}^{\infty } {\tau^{n^\circ + c} \,\gamma^{n^\circ + a - 1} e^{ - (b\gamma + d\tau )} \left[ {\sum\limits_{{l_{1}^{1} = 0}}^{0} {\;\sum\limits_{{l_{2}^{1} = 0}}^{1} {...\sum\limits_{{l_{m}^{1} = 0}}^{m - 1} {} } } } \right]\left[ {\sum\limits_{{l_{1}^{2} = 0}}^{0} {\;\sum\limits_{{l_{2}^{2} = 0}}^{1} {...\sum\limits_{{l_{m}^{2} = 0}}^{m - 1} {} } } } \right]\;...\;\left[ {\sum\limits_{{l_{1}^{n} = 0}}^{0} {\;\sum\limits_{{l_{2}^{n} = 0}}^{1} {...\sum\limits_{{l_{m}^{n} = 0}}^{m - 1} {} } } } \right]\left[ {\prod\limits_{j = 1}^{n} {\prod\limits_{i = 1}^{m} {V_{{l_{i}^{j} }} } } } \right]d\gamma d\tau } } }}{{\int\limits_{0}^{\infty } {\int\limits_{0}^{\infty } {\tau^{n^\circ + c - 1} \,\gamma^{n^\circ + a - 1} e^{ - (b\gamma + d\tau )} \left[ {\sum\limits_{{l_{1}^{1} = 0}}^{0} {\;\sum\limits_{{l_{2}^{1} = 0}}^{1} {...\sum\limits_{{l_{m}^{1} = 0}}^{m - 1} {} } } } \right]\left[ {\sum\limits_{{l_{1}^{2} = 0}}^{0} {\;\sum\limits_{{l_{2}^{2} = 0}}^{1} {...\sum\limits_{{l_{m}^{2} = 0}}^{m - 1} {} } } } \right]\;...\;\left[ {\sum\limits_{{l_{1}^{n} = 0}}^{0} {\;\sum\limits_{{l_{2}^{n} = 0}}^{1} {...\sum\limits_{{l_{m}^{n} = 0}}^{m - 1} {} } } } \right]\left[ {\prod\limits_{j = 1}^{n} {\prod\limits_{i = 1}^{m} {V_{{l_{i}^{j} }} } } } \right]d\gamma d\tau } } }}.$$

ii. Bayesian estimators under MELoF

The MELoF is contributed by Tummala and Sathe^44^, and it is believed to be a special case of the commonly used quadratic LoFs, which is defined by:

14 $$L_{MELoF} (\beta ,\tilde{\beta }) = \varphi (\tilde{\beta } - \beta )_{{}}^{2} . \,$$

If $\varphi = 1$ in Eq. 14, then it reduces to SELoF, and for $\varphi = \beta_{{}}^{ - 2}$ it becomes MELoF

$$L_{MELoF} (\beta ,\tilde{\beta }) = \varphi_{{}}^{ - 2} \,(\tilde{\beta } - \beta )^{2} .$$

Based on MELoF, the BE of the unknown parameter $\beta$ is given by

15 $$\tilde{\beta }_{MELoF} = \frac{{E(\beta^{ - 1} \left| {\underline{x} } \right.)}}{{E(\beta^{ - 2} \left| {\underline{x} } \right.)}} = \frac{{\int\limits_{\beta } {\beta^{ - 1} \;w(\beta \left| {\underline{x} } \right.)} \;d\beta }}{{\int\limits_{\beta } {\beta^{ - 2} \;w(\beta \left| {\underline{x} } \right.)} \;d\beta }},$$

where $\tilde{\beta }_{MELoF}$ is the BE for $\beta=(\gamma, \tau)$ under MELoF. Considering Eq. 15, the BE for $\gamma$ under MELoF, denoted by $\tilde{\gamma }_{MELoF} ,$ is obtained as follows:

$$\tilde{\gamma }_{MELoF} = \frac{{E(\gamma^{ - 1} \left| {\underline{x} } \right.)}}{{E(\gamma^{ - 2} \left| {\underline{x} } \right.)}} = \frac{{\int\limits_{\gamma } {\gamma^{ - 1} \;h_{1} (\gamma \left| {\underline{x} } \right.)} \;d\gamma }}{{\int\limits_{\gamma } {\gamma^{ - 2} \;h_{1} (\gamma \left| {\underline{x} } \right.)} \;d\gamma }}.$$

Hence,

16 $$\tilde{\gamma }_{MELoF} = \frac{{\int\limits_{0}^{\infty } {\int\limits_{{0}}^{\infty } {\gamma^{n^\circ + a - 2} e^{ - (b\gamma + d\tau )} \tau^{n^\circ + c - 1} \left[ {\sum\limits_{{l_{1}^{1} = 0}}^{0} {\;\sum\limits_{{l_{2}^{1} = 0}}^{1} {...\sum\limits_{{l_{m}^{1} = 0}}^{m - 1} {} } } } \right]\left[ {\sum\limits_{{l_{1}^{2} = 0}}^{0} {\;\sum\limits_{{l_{2}^{2} = 0}}^{1} {...\sum\limits_{{l_{m}^{2} = 0}}^{m - 1} {} } } } \right]\;...\;\left[ {\sum\limits_{{l_{1}^{n} = 0}}^{0} {\;\sum\limits_{{l_{2}^{n} = 0}}^{1} {...\sum\limits_{{l_{m}^{n} = 0}}^{m - 1} {} } } } \right]\left[ {\prod\limits_{j = 1}^{n} {\prod\limits_{i = 1}^{m} {V_{{l_{i}^{j} }} } } } \right]\;d\tau \,} } d\gamma }}{{\int\limits_{0}^{\infty } {\int\limits_{{0}}^{\infty } {\gamma^{n^\circ + a - 3} e^{ - (b\gamma + d\tau )} \tau^{n^\circ + c - 1} \left[ {\sum\limits_{{l_{1}^{1} = 0}}^{0} {\;\sum\limits_{{l_{2}^{1} = 0}}^{1} {...\sum\limits_{{l_{m}^{1} = 0}}^{m - 1} {} } } } \right]\left[ {\sum\limits_{{l_{1}^{2} = 0}}^{0} {\;\sum\limits_{{l_{2}^{2} = 0}}^{1} {...\sum\limits_{{l_{m}^{2} = 0}}^{m - 1} {} } } } \right]\;...\;\left[ {\sum\limits_{{l_{1}^{n} = 0}}^{0} {\;\sum\limits_{{l_{2}^{n} = 0}}^{1} {...\sum\limits_{{l_{m}^{n} = 0}}^{m - 1} {} } } } \right]\left[ {\prod\limits_{j = 1}^{n} {\prod\limits_{i = 1}^{m} {V_{{l_{i}^{j} }} } } } \right]\;d\tau \,} } d\gamma }}.$$

In a similar manner, the BE of $\tau$ under the MELoF, say $\tilde{\tau }_{MELoF} ,$ is obtained as follows:

17 $$\tilde{\tau }_{MELoF} = \frac{{\int\limits_{0}^{\infty } {\int\limits_{0}^{\infty } {\gamma^{n^\circ + a - 1} e^{ - (b\gamma + d\tau )} \tau^{n^\circ + c - 2} \left[ {\sum\limits_{{l_{1}^{1} = 0}}^{0} {\;\sum\limits_{{l_{2}^{1} = 0}}^{1} {...\sum\limits_{{l_{m}^{1} = 0}}^{m - 1} {} } } } \right]\left[ {\sum\limits_{{l_{1}^{2} = 0}}^{0} {\;\sum\limits_{{l_{2}^{2} = 0}}^{1} {...\sum\limits_{{l_{m}^{2} = 0}}^{m - 1} {} } } } \right]\;...\;\left[ {\sum\limits_{{l_{1}^{n} = 0}}^{0} {\;\sum\limits_{{l_{2}^{n} = 0}}^{1} {...\sum\limits_{{l_{m}^{n} = 0}}^{m - 1} {} } } } \right]\left[ {\prod\limits_{j = 1}^{n} {\prod\limits_{i = 1}^{m} {V_{{l_{i}^{j} }} } } } \right]d\gamma \,d\tau } } }}{{\int\limits_{0}^{\infty } {\int\limits_{0}^{\infty } {\gamma^{n^\circ + a - 1} e^{ - (b\gamma + d\tau )} \tau^{n^\circ + c - 3} \left[ {\sum\limits_{{l_{1}^{1} = 0}}^{0} {\;\sum\limits_{{l_{2}^{1} = 0}}^{1} {...\sum\limits_{{l_{m}^{1} = 0}}^{m - 1} {} } } } \right]\left[ {\sum\limits_{{l_{1}^{2} = 0}}^{0} {\;\sum\limits_{{l_{2}^{2} = 0}}^{1} {...\sum\limits_{{l_{m}^{2} = 0}}^{m - 1} {} } } } \right]\;...\;\left[ {\sum\limits_{{l_{1}^{n} = 0}}^{0} {\;\sum\limits_{{l_{2}^{n} = 0}}^{1} {...\sum\limits_{{l_{m}^{n} = 0}}^{m - 1} {} } } } \right]\left[ {\prod\limits_{j = 1}^{n} {\prod\limits_{i = 1}^{m} {V_{{l_{i}^{j} }} } } } \right]d\gamma \,d\tau } } }}.$$

Since these integrals in Eqs. 12, 13, 16, and 17 have no closed form, they cannot be solved analytically. Therefore, under SELoF and MELoF, the BEs of $\gamma ,$ and $\tau$ will be obtained using the MH technique. In the case of NIFP, the previous BEs are achieved by putting the hyper-parameters *a* = *b* = *c* = *d* = 0.0001 in Eq. 8 and then using the same MH technique.

## Estimation methods based on MRSSU

The IKmD parameters based on MRSSU are estimated using non-Bayesian and Bayesian approaches in this section.

### Maximum likelihood estimators

Let (*Y*_*ij,*_* i* = 1,2,…,*m*, *j* = 1,2,…,*n* ) denote the MRSSU of size $n^\circ = mn$ from the IKmD with parameters $\gamma ,\tau > 0$, where the set size is *m* and the cycle count is *n*. According to Tahmasebi et al.^45^, the PDF of *Y*_*ij*_ under the assumption of perfect ranking, is exactly the PDF of *i*th order statistics given by:

18 $$f(y_{ij} ) = \,i\;{\kern 1pt} f( \, y_{(ij)} ;\gamma ,\tau )\left[ {F(y_{(ij)} ;\gamma ,\tau )} \right]^{i - 1} .\quad \;\quad \quad y,\;\gamma ,\;\tau > 0,$$

Then the likelihood function is provided by using CDF (1) and PDF (2) in Eq. 18

19 $$L_{2} (\left. {\underline{y} } \right|\gamma ,\tau ) = \,{\kern 1pt} \tau^{n^\circ } \,\gamma^{n^\circ } \prod\limits_{j = 1}^{n} {\prod\limits_{i = 1}^{m} {i\;\left( {1 + y_{ij} } \right)^{ - \gamma - 1} \left[ {1 - {\kern 1pt} (1 + y_{ij} ){\kern 1pt}^{ - \gamma } {\kern 1pt} } \right]^{i\tau - 1} \;\,} } ,$$

then the log-likelihood function is

20 $$l_{2} (\left. {\underline{y} } \right|\gamma ,\tau )\, \propto n^\circ \,\ln \,\tau_{{}}^{{}} + n^\circ \,\ln \,\gamma_{{}}^{{}} - (\gamma + 1)\sum\limits_{j = 1}^{n} {\sum\limits_{i = 1}^{m} {\ln } \left( {1 + y_{ij} } \right)} + \sum\limits_{j = 1}^{n} {\sum\limits_{i = 1}^{m} {(i\tau - 1)\ln \left[ {1 - {\kern 1pt} {\kern 1pt} (1 + y_{ij} ){\kern 1pt}^{ - \gamma } } \right]\,} } {.}$$

The partial differential equations of $l_{2} (\left. {\underline{y} } \right|\gamma ,\tau )$ with respect to $\gamma ,$ and $\tau$ are given, respectively, by:

21 $$\frac{{\partial \;l_{{_{2} }}^{{}} (\left. {\underline{y} } \right|\gamma ,\tau )}}{\partial \gamma }{\kern 1pt} = \,\frac{n^\circ }{\gamma } - \sum\limits_{j = 1}^{n} {\sum\limits_{i = 1}^{m} {\ln } \left( {1 + y_{ij} } \right)} \; + \sum\limits_{j = 1}^{n} {\sum\limits_{i = 1}^{m} {\frac{{(i\tau - 1)\;(1 + y_{ij} ){\kern 1pt}^{ - \gamma } \;\ln (1 + y_{ij} )\;}}{{\left[ {1 - {\kern 1pt} {\kern 1pt} (1 + y_{ij} ){\kern 1pt}^{ - \gamma } } \right]}}\,} } ,$$

and,

22 $$\frac{{\partial \;l_{2} (\left. {\underline{y} } \right|\gamma ,\tau )}}{\partial \tau }{\kern 1pt} = \,\frac{n^\circ }{\tau } + \sum\limits_{j = 1}^{n} {\sum\limits_{i = 1}^{m} {i\ln \left[ {1 - {\kern 1pt} {\kern 1pt} (1 + y_{ij} ){\kern 1pt}^{ - \gamma } } \right]\,} .}$$

We can get the MLE for parameters $\gamma ,$ and $\tau$ say $\hat{\gamma }_{2} ,$ and $\hat{\tau }_{2}$ after maximizing Eq. 20 for the log likelihood function or by equating Eqs. 21 and 22 to zero and solving numerically.

$$\left. {\frac{{\partial \;l_{2} (\left. {\underline{y} } \right|\gamma ,\tau )}}{\partial \gamma }} \right|_{{\gamma = \hat{\gamma }_{2} }} {\kern 1pt} = 0,\;\;\text{and}\;\;\left. {\frac{{\partial \;l_{2} (\left. {\underline{y} } \right|\gamma ,\tau )}}{\partial \tau }} \right|_{{\tau = \hat{\tau }_{2} }} {\kern 1pt} = 0.$$

These equations are difficult to solve in closed form; hence, numerical methods must be used to solve them by using package “bbmle” in the R 4.3.0 program.

### Bayes estimation based on MRSSU

The estimators of the parameters of the IKmD based on MRSSU are obtained using the Bayesian approach. The IFP and NIFP, represented by gamma and uniform priors, respectively, were used to derive BE. The BEs in the case of IFP can be calculated by using the same independent gamma priors utilized in Eq. 8. Using binomial expansion, the last term of Eq. 19 can be written as:

$$\left[ {1 - {\kern 1pt} (1 + y_{ij} ){\kern 1pt}^{ - \gamma } {\kern 1pt} } \right]^{i\tau - 1} = \sum\limits_{{w_{i} = 0}}^{\infty } {\left( {\begin{array}{*{20}c} {i\tau - 1} \\ {w_{i} } \\ \end{array} } \right)\left( { - 1} \right)^{{w_{i} }} } (1 + y_{i} ){\kern 1pt}^{{ - \gamma \,w_{i} }} .$$

Then, Eq. 19 for the likelihood function can be written as:

23 $$L_{2} (\left. {\gamma ,\tau } \right|\underline{y} ) = \,\gamma^{n^\circ } \;\tau^{n^\circ } {\kern 1pt} \left[ {\sum\limits_{{w_{1}^{1} = 0}}^{0} {\;\sum\limits_{{w_{2}^{1} = 0}}^{1} {...\sum\limits_{{w_{m}^{1} = 0}}^{m - 1} {} } } } \right]\left[ {\sum\limits_{{w_{1}^{2} = 0}}^{0} {\;\sum\limits_{{w_{2}^{2} = 0}}^{1} {...\sum\limits_{{w_{m}^{2} = 0}}^{m - 1} {} } } } \right]\;...\;\left[ {\sum\limits_{{w_{1}^{n} = 0}}^{0} {\;\sum\limits_{{w_{2}^{n} = 0}}^{1} {...\sum\limits_{{w_{m}^{n} = 0}}^{m - 1} {} } } } \right]\left[ {\prod\limits_{j = 1}^{n} {\prod\limits_{i = 1}^{m} {R_{{w_{i}^{j} }} } } } \right],$$

where $$R_{{w_{i}^{j} }} = \left( {z_{{w_{i} }} (i){\kern 1pt} \left( {1 + y_{i} } \right)^{{ - \gamma \;w_{i} - \gamma - 1}} } \right),$$ and $$z_{{w_{i} }} (i) = i\left( {\begin{array}{*{20}c} {i\tau - 1} \\ {w_{i} } \\ \end{array} } \right)\left( { - 1} \right)^{{w_{i} }} .$$

Based on Eqs. 8 and 23 for the joint prior distribution of the parameters and the likelihood function, respectively, the joint posterior density of $\gamma ,$ and $\tau$ given the data $\underline{y}$ can be written as follows:

24 $$\pi_{2} (\left. {\gamma ,\tau } \right|\underline{y} \;) = \frac{{L_{2} (\left. {\gamma ,\tau } \right|\underline{y} \;)\;\;g_{1,2} (\gamma ,\tau )}}{{\int\limits_{\gamma } {\int\limits_{\tau } {L_{2} (\left. {\gamma ,\tau } \right|\underline{y} \;)\;\;g_{1,2} (\gamma ,\tau )\;d\tau \;d\gamma } } }}.$$

From Eqs. 8, 23, and 24, the joint posterior for $(\gamma ,\tau )$ can be written as follows:

$$\pi_{2} (\left. {\gamma ,\tau } \right|\underline{y} \;) \propto \;\gamma^{n^\circ + a - 1} \tau^{n^\circ + c - 1} e^{ - b\gamma - d\tau } {\kern 1pt} \left[ {\sum\limits_{{w_{1}^{1} = 0}}^{0} {\;\sum\limits_{{w_{2}^{1} = 0}}^{1} {...\sum\limits_{{w_{m}^{1} = 0}}^{m - 1} {} } } } \right]\left[ {\sum\limits_{{w_{1}^{2} = 0}}^{0} {\;\sum\limits_{{w_{2}^{2} = 0}}^{1} {...\sum\limits_{{w_{m}^{2} = 0}}^{m - 1} {} } } } \right]\;...\;\left[ {\sum\limits_{{w_{1}^{n} = 0}}^{0} {\;\sum\limits_{{w_{2}^{n} = 0}}^{1} {...\sum\limits_{{w_{m}^{n} = 0}}^{m - 1} {} } } } \right]\left[ {\prod\limits_{j = 1}^{n} {\prod\limits_{i = 1}^{m} {{\text{R}}_{{w_{{\text{i}}}^{{\text{j}}} }} } } } \right].$$

Hence, the conditional posterior densities for $\gamma ,$ and $\tau$ take the following forms

$$h_{3} (\left. \gamma \right|\tau,\underline{y} ) = K_{2}^{ - 1} \;\gamma^{n^\circ + a - 1} \;e^{ - b\gamma } \int\limits_{{0}}^{\infty } {\tau^{n^\circ + c - 1} e^{ - d\tau } {\kern 1pt} \left[ {\sum\limits_{{w_{1}^{1} = 0}}^{0} {\;\sum\limits_{{w_{2}^{1} = 0}}^{1} {...\sum\limits_{{w_{m}^{1} = 0}}^{m - 1} {} } } } \right]\left[ {\sum\limits_{{w_{1}^{2} = 0}}^{0} {\;\sum\limits_{{w_{2}^{2} = 0}}^{1} {...\sum\limits_{{w_{m}^{2} = 0}}^{m - 1} {} } } } \right]\;...\;\left[ {\sum\limits_{{w_{1}^{n} = 0}}^{0} {\;\sum\limits_{{w_{2}^{n} = 0}}^{1} {...\sum\limits_{{w_{m}^{n} = 0}}^{m - 1} {} } } } \right]\left[ {\prod\limits_{j = 1}^{n} {\prod\limits_{i = 1}^{m} {{\text{R}}_{{w_{{\text{i}}}^{{\text{j}}} }} } } } \right]\;d\tau ,}$$

and,

$$h_{4} (\left. \tau \right|\gamma,\underline{y} ) = K_{2}^{ - 1} \;\tau^{n^\circ + c - 1} e^{ - d\tau } \int\limits_{{0}}^{\infty } {\;\gamma^{n^\circ + a - 1} e^{ - b\gamma } {\kern 1pt} \left[ {\sum\limits_{{w_{1}^{1} = 0}}^{0} {\;\sum\limits_{{w_{2}^{1} = 0}}^{1} {...\sum\limits_{{w_{m}^{1} = 0}}^{m - 1} {} } } } \right]\left[ {\sum\limits_{{w_{1}^{2} = 0}}^{0} {\;\sum\limits_{{w_{2}^{2} = 0}}^{1} {...\sum\limits_{{w_{m}^{2} = 0}}^{m - 1} {} } } } \right]\;...\;\left[ {\sum\limits_{{w_{1}^{n} = 0}}^{0} {\;\sum\limits_{{w_{2}^{n} = 0}}^{1} {...\sum\limits_{{w_{m}^{n} = 0}}^{m - 1} {} } } } \right]\left[ {\prod\limits_{j = 1}^{n} {\prod\limits_{i = 1}^{m} {{\text{R}}_{{w_{{\text{i}}}^{{\text{j}}} }} } } } \right]\;d\gamma } ,$$

where

$$K_{2} = \int\limits_{0}^{\infty } {\int\limits_{0}^{\infty } {\gamma^{n^\circ + a - 1} \tau^{n^\circ + c - 1} e^{ - b\gamma - d\tau } \left[ {\sum\limits_{{w_{1}^{1} = 0}}^{0} {\;\sum\limits_{{w_{2}^{1} = 0}}^{1} {...\sum\limits_{{w_{m}^{1} = 0}}^{m - 1} {} } } } \right]\left[ {\sum\limits_{{w_{1}^{2} = 0}}^{0} {\;\sum\limits_{{w_{2}^{2} = 0}}^{1} {...\sum\limits_{{w_{m}^{2} = 0}}^{m - 1} {} } } } \right]\;...\;\left[ {\sum\limits_{{w_{1}^{n} = 0}}^{0} {\;\sum\limits_{{w_{2}^{n} = 0}}^{1} {...\sum\limits_{{w_{m}^{n} = 0}}^{m - 1} {} } } } \right]\left[ {\prod\limits_{j = 1}^{n} {\prod\limits_{i = 1}^{m} {{\text{R}}_{{w_{{\text{i}}}^{{\text{j}}} }} } } } \right]d\tau \;d\gamma } .}$$

Since the distributions of these marginal posterior distributions are unknown, we can handle them by applying the MH approach.

- Bayesian estimators under SELoF

Here, the BEs of $$\gamma ,$$ and $$\tau$$ under SELoF are derived. Hence, based on Eq. 11, the BE of $$\gamma$$ under SELoF, denoted by $$\overset{\lower0.5em\hbox{$\smash{\scriptscriptstyle\frown}$}}{\gamma }_{SELoF} ,$$ can be obtained as

25 $$\overset{\lower0.5em\hbox{$\smash{\scriptscriptstyle\frown}$}}{\gamma }_{SELoF} = \frac{{\int\limits_{{0}}^{\infty } {\int\limits_{{0}}^{\infty } {\gamma^{n^\circ + a} e^{ - (b\gamma + d\tau )} \tau^{n^\circ + c - 1} {\kern 1pt} \left[ {\sum\limits_{{w_{1}^{1} = 0}}^{0} {\;\sum\limits_{{w_{2}^{1} = 0}}^{1} {...\sum\limits_{{w_{m}^{1} = 0}}^{m - 1} {} } } } \right]\left[ {\sum\limits_{{w_{1}^{2} = 0}}^{0} {\;\sum\limits_{{w_{2}^{2} = 0}}^{1} {...\sum\limits_{{w_{m}^{2} = 0}}^{m - 1} {} } } } \right]\;...\;\left[ {\sum\limits_{{w_{1}^{n} = 0}}^{0} {\;\sum\limits_{{w_{2}^{n} = 0}}^{1} {...\sum\limits_{{w_{m}^{n} = 0}}^{m - 1} {} } } } \right]\left[ {\prod\limits_{j = 1}^{n} {\prod\limits_{i = 1}^{m} {{\text{R}}_{{w_{{\text{i}}}^{{\text{j}}} }} } } } \right]\;d\tau } d\gamma } }}{{\;\;\int\limits_{{0}}^{\infty } {\int\limits_{{0}}^{\infty } {\gamma^{n^\circ + a - 1} e^{ - (b\gamma + d\tau )} \tau^{n^\circ + c - 1} {\kern 1pt} \left[ {\sum\limits_{{w_{1}^{1} = 0}}^{0} {\;\sum\limits_{{w_{2}^{1} = 0}}^{1} {...\sum\limits_{{w_{m}^{1} = 0}}^{m - 1} {} } } } \right]\left[ {\sum\limits_{{w_{1}^{2} = 0}}^{0} {\;\sum\limits_{{w_{2}^{2} = 0}}^{1} {...\sum\limits_{{w_{m}^{2} = 0}}^{m - 1} {} } } } \right]\;...\;\left[ {\sum\limits_{{w_{1}^{n} = 0}}^{0} {\;\sum\limits_{{w_{2}^{n} = 0}}^{1} {...\sum\limits_{{w_{m}^{n} = 0}}^{m - 1} {} } } } \right]\left[ {\prod\limits_{j = 1}^{n} {\prod\limits_{i = 1}^{m} {{\text{R}}_{{w_{{\text{i}}}^{{\text{j}}} }} } } } \right]\;d\tau } d\gamma } }}.$$

Similarly, the BE of $$\tau$$ under the SELoF, say $$\overset{\lower0.5em\hbox{$\smash{\scriptscriptstyle\frown}$}}{\tau }_{SELoF}$$ is given by:

26 $$\overset{\lower0.5em\hbox{$\smash{\scriptscriptstyle\frown}$}}{\tau }_{SELoF} = \frac{{\int\limits_{0}^{\infty } {\int\limits_{{0}}^{\infty } {\gamma^{n^\circ + a - 1} e^{ - (b\gamma + d\tau )} \tau^{n^\circ + c} {\kern 1pt} \left[ {\sum\limits_{{w_{1}^{1} = 0}}^{0} {\;\sum\limits_{{w_{2}^{1} = 0}}^{1} {...\sum\limits_{{w_{m}^{1} = 0}}^{m - 1} {} } } } \right]\left[ {\sum\limits_{{w_{1}^{2} = 0}}^{0} {\;\sum\limits_{{w_{2}^{2} = 0}}^{1} {...\sum\limits_{{w_{m}^{2} = 0}}^{m - 1} {} } } } \right]\;...\;\left[ {\sum\limits_{{w_{1}^{n} = 0}}^{0} {\;\sum\limits_{{w_{2}^{n} = 0}}^{1} {...\sum\limits_{{w_{m}^{n} = 0}}^{m - 1} {} } } } \right]\left[ {\prod\limits_{j = 1}^{n} {\prod\limits_{i = 1}^{m} {{\text{R}}_{{w_{{\text{i}}}^{{\text{j}}} }} } } } \right]\;d\gamma d\tau } } }}{{\int\limits_{0}^{\infty } {\int\limits_{{0}}^{\infty } {\gamma^{n^\circ + a - 1} e^{ - (b\gamma + d\tau )} \tau^{n^\circ + c - 1} {\kern 1pt} \left[ {\sum\limits_{{w_{1}^{1} = 0}}^{0} {\;\sum\limits_{{w_{2}^{1} = 0}}^{1} {...\sum\limits_{{w_{m}^{1} = 0}}^{m - 1} {} } } } \right]\left[ {\sum\limits_{{w_{1}^{2} = 0}}^{0} {\;\sum\limits_{{w_{2}^{2} = 0}}^{1} {...\sum\limits_{{w_{m}^{2} = 0}}^{m - 1} {} } } } \right]\;...\;\left[ {\sum\limits_{{w_{1}^{n} = 0}}^{0} {\;\sum\limits_{{w_{2}^{n} = 0}}^{1} {...\sum\limits_{{w_{m}^{n} = 0}}^{m - 1} {} } } } \right]\left[ {\prod\limits_{j = 1}^{n} {\prod\limits_{i = 1}^{m} {{\text{R}}_{{w_{{\text{i}}}^{{\text{j}}} }} } } } \right]\;d\gamma d\tau } } }}.$$

b. Bayesian estimators under MELoF

Here, the BEs for $$\gamma ,$$ and $$\tau$$ under MELoF are obtained. Using Eq. 15, the BE of $$\gamma$$ under MELoF, denoted by $$\overset{\lower0.5em\hbox{$\smash{\scriptscriptstyle\frown}$}}{\gamma }_{MELoF} ,$$ is obtained as follows:

27 $$\overset{\lower0.5em\hbox{$\smash{\scriptscriptstyle\frown}$}}{\gamma }_{MELoF} = \frac{{\int\limits_{0}^{\infty } {\int\limits_{{0}}^{\infty } {\gamma^{n^\circ + a - 2} e^{ - (b\gamma + d\tau )} \tau^{n^\circ + c - 1} {\kern 1pt} \left[ {\sum\limits_{{w_{1}^{1} = 0}}^{0} {\;\sum\limits_{{w_{2}^{1} = 0}}^{1} {...\sum\limits_{{w_{m}^{1} = 0}}^{m - 1} {} } } } \right]\left[ {\sum\limits_{{w_{1}^{2} = 0}}^{0} {\;\sum\limits_{{w_{2}^{2} = 0}}^{1} {...\sum\limits_{{w_{m}^{2} = 0}}^{m - 1} {} } } } \right]\;...\;\left[ {\sum\limits_{{w_{1}^{n} = 0}}^{0} {\;\sum\limits_{{w_{2}^{n} = 0}}^{1} {...\sum\limits_{{w_{m}^{n} = 0}}^{m - 1} {} } } } \right]\left[ {\prod\limits_{j = 1}^{n} {\prod\limits_{i = 1}^{m} {{\text{R}}_{{w_{{\text{i}}}^{{\text{j}}} }} } } } \right]\;d\tau \,} } d\gamma }}{{\int\limits_{0}^{\infty } {\int\limits_{{0}}^{\infty } {\gamma^{n^\circ + a - 3} e^{ - (b\gamma + d\tau )} \tau^{n^\circ + c - 1} {\kern 1pt} \left[ {\sum\limits_{{w_{1}^{1} = 0}}^{0} {\;\sum\limits_{{w_{2}^{1} = 0}}^{1} {...\sum\limits_{{w_{m}^{1} = 0}}^{m - 1} {} } } } \right]\left[ {\sum\limits_{{w_{1}^{2} = 0}}^{0} {\;\sum\limits_{{w_{2}^{2} = 0}}^{1} {...\sum\limits_{{w_{m}^{2} = 0}}^{m - 1} {} } } } \right]\;...\;\left[ {\sum\limits_{{w_{1}^{n} = 0}}^{0} {\;\sum\limits_{{w_{2}^{n} = 0}}^{1} {...\sum\limits_{{w_{m}^{n} = 0}}^{m - 1} {} } } } \right]\left[ {\prod\limits_{j = 1}^{n} {\prod\limits_{i = 1}^{m} {{\text{R}}_{{w_{{\text{i}}}^{{\text{j}}} }} } } } \right]\;d\tau \,} } d\gamma }}.$$

In a similar manner, the BE of $$\tau$$ under the MELoF, say $$\overset{\lower0.5em\hbox{$\smash{\scriptscriptstyle\frown}$}}{\tau }_{MELoF} ,$$ is obtained as follows:

28 $$\overset{\lower0.5em\hbox{$\smash{\scriptscriptstyle\frown}$}}{\tau }_{MELoF} = \frac{{\int\limits_{0}^{\infty } {\int\limits_{{0}}^{\infty } {\gamma^{n^\circ + a - 1} e^{ - (b\gamma + d\tau )} \tau^{n^\circ + c - 2} {\kern 1pt} \left[ {\sum\limits_{{w_{1}^{1} = 0}}^{0} {\;\sum\limits_{{w_{2}^{1} = 0}}^{1} {...\sum\limits_{{w_{m}^{1} = 0}}^{m - 1} {} } } } \right]\left[ {\sum\limits_{{w_{1}^{2} = 0}}^{0} {\;\sum\limits_{{w_{2}^{2} = 0}}^{1} {...\sum\limits_{{w_{m}^{2} = 0}}^{m - 1} {} } } } \right]\;...\;\left[ {\sum\limits_{{w_{1}^{n} = 0}}^{0} {\;\sum\limits_{{w_{2}^{n} = 0}}^{1} {...\sum\limits_{{w_{m}^{n} = 0}}^{m - 1} {} } } } \right]\left[ {\prod\limits_{j = 1}^{n} {\prod\limits_{i = 1}^{m} {{\text{R}}_{{w_{{\text{i}}}^{{\text{j}}} }} } } } \right]\;d\gamma \,d\tau } } }}{{\int\limits_{0}^{\infty } {\int\limits_{{0}}^{\infty } {\gamma^{n^\circ + a - 1} e^{ - (b\gamma + d\tau )} \tau^{n^\circ + c - 3} {\kern 1pt} \left[ {\sum\limits_{{w_{1}^{1} = 0}}^{0} {\;\sum\limits_{{w_{2}^{1} = 0}}^{1} {...\sum\limits_{{w_{m}^{1} = 0}}^{m - 1} {} } } } \right]\left[ {\sum\limits_{{w_{1}^{2} = 0}}^{0} {\;\sum\limits_{{w_{2}^{2} = 0}}^{1} {...\sum\limits_{{w_{m}^{2} = 0}}^{m - 1} {} } } } \right]\;...\;\left[ {\sum\limits_{{w_{1}^{n} = 0}}^{0} {\;\sum\limits_{{w_{2}^{n} = 0}}^{1} {...\sum\limits_{{w_{m}^{n} = 0}}^{m - 1} {} } } } \right]\left[ {\prod\limits_{j = 1}^{n} {\prod\limits_{i = 1}^{m} {{\text{R}}_{{w_{{\text{i}}}^{{\text{j}}} }} } } } \right]\;d\gamma \,d\tau } } }}.$$

Since these integrals in Eqs. 25, 26, 27, and 28 are not in a closed form, they are unable to be calculated analytically. Therefore, BEs for $\gamma ,$ and $\tau$ under SELoF and MELoF will be obtained using the MH technique. The previous BEs in the case of NIFP are obtained by substituting the hyper-parameters *a* = *b* = *c* = *d* = 0.0001 in Eq. 8 and applying the same MH approach.

## Simulation results

Simulation research is conducted to assess how well the estimate methods function under RSS and MRSSU. The IKmD was used to create random samples for various values for $\gamma ,$ and $\tau .$ This study is conducted to investigate and contrast the MLE and various BE behaviours for the IKmD. The BEs are derived using gamma and Jeffreys priors under SELoF and MELoF.

### Monto Carlo simulation

The simulation algorithm is as follows:

1. From the IKmD, create MRSSU and RSS of size *m*.
2. Compute the MLE and BE derived in Sect. “Estimation methods based on RSS” and “Estimation methods based on MRSSU” for RSS and MRSSU, respectively, for each set of parameters using various numbers of set sizes *m* = 2, 3, 4, and 5 and using package “bbmle” for the maximum likelihood method.
3. Repeat steps 1 and 2 for *n* = 5 and 10 cycles to obtain a sample of sizes $n^\circ$ = 10, 15, 20, 25, 30, 40, and 50.
4. For the purpose of generating random samples, the following parameter values are chosen as: $(i)\;\gamma = 0.5\;,\;\tau = 0.8, \, (ii)\;\gamma = 0.5\;,\;\tau = 1.2, \, (iii)\;\gamma = 1.5\;,\;\tau = 1.5$.
5. Following Dey et al.^46^, the hyper-parameters for gamma priors are determined by equating the variance and mean for estimates with the variance and mean for the gamma distribution.
6. To perform the MH algorithm, define a proposal distribution $H(\beta^{\prime}\;\left| \beta \right.)$ and initial values $\beta^{(0)}$ of the unknown parameters. For the proposal distribution, consider a bivariate normal distribution, that is, take $H(\beta^{\prime}\;\left| \beta \right.) = N_{2} (\beta ,S_{\beta }^{ \cdot } )$ where $\beta = (\gamma ,\tau )$ and $S_{\beta }^{ \cdot }$ represents the variance covariance matrix. It is to be noted that the bivariate normal distribution may generate negative observations that are unacceptable. The initial value for $\beta$ is the MLE of $\beta$. The selection of $S_{\beta }^{ \cdot }$ is an important issue in the MH algorithm, where the acceptance rate depends on this (see Dey and Pradhan^47^). The previous steps are done via R 4.3.0 program.
  The following steps of the MH algorithm are used to draw a sample from the posterior density:
  - Set the initial value of $\beta$ as $\beta = \beta^{(0)}$.
  - For *i* = 1,2,…, *M* repeat the following steps:
    - Set $$\beta = \beta^{(i - 1)}$$.
    - Generate a new candidate parameter value $\vartheta$ from $N_{2} (\ln \beta ,S_{\beta }^{ \cdot } )$.
    - Set $$\beta^{\prime} = \exp (\vartheta ).$$
    - Calculate $$\varepsilon = \min \left[ {1,\frac{{\pi (\beta^{\prime}\left| x \right.)\;\beta^{\prime}}}{\pi (\beta \left| x \right.)\;\beta }} \right].$$
    - Generate a sample *u* from the uniform *U* (0, 1) distribution.
    - Accept or reject the new candidate $\beta^{\prime}$
      $$\left\{ {\begin{array}{*{20}c} {If \, u < \varepsilon {\text{ Set }}\beta^{(i)} = \beta^{\prime}} \\ {{\text{otherwise Set }}\beta^{(i)} = \beta .} \\ \end{array} } \right.$$
  Some of the early samples (burn-in) can be eliminated from the random samples of size M selected from the posterior density, and the remaining samples can then be processed further to determine BE. The BE depending on the SELoF is supplied by:
  $$\tilde{g}_{MH} (\beta ) = \frac{1}{{M - \iota_{B} }}\sum\limits_{{i = \iota_{B} }}^{M} {g(\beta )} {\kern 1pt} ,$$
  where $\iota_{B}$ represents the number of burn-in samples.
  7. Finally, out of the 10,000 samples that the posterior density produced in total, we eliminated 2000 samples that were burn-in.
  8. The RAB and RMSE are used to compare results for different samples.

$$RAB = \frac{1}{N}\sum\limits_{i = 1}^{N} {\left| {\frac{{\hat{\beta }_{i} - \beta }}{\beta }} \right|} {, }\;\;\text{and}\;\;RMSE{ = }\sqrt {\frac{1}{N}\sum\limits_{i = 1}^{N} {(\hat{\beta }_{i} - \beta )^{2} } } ,$$

where, *N* = 10,000 is the number of re-samplings.

### Numerical results

**Fig. 4:**
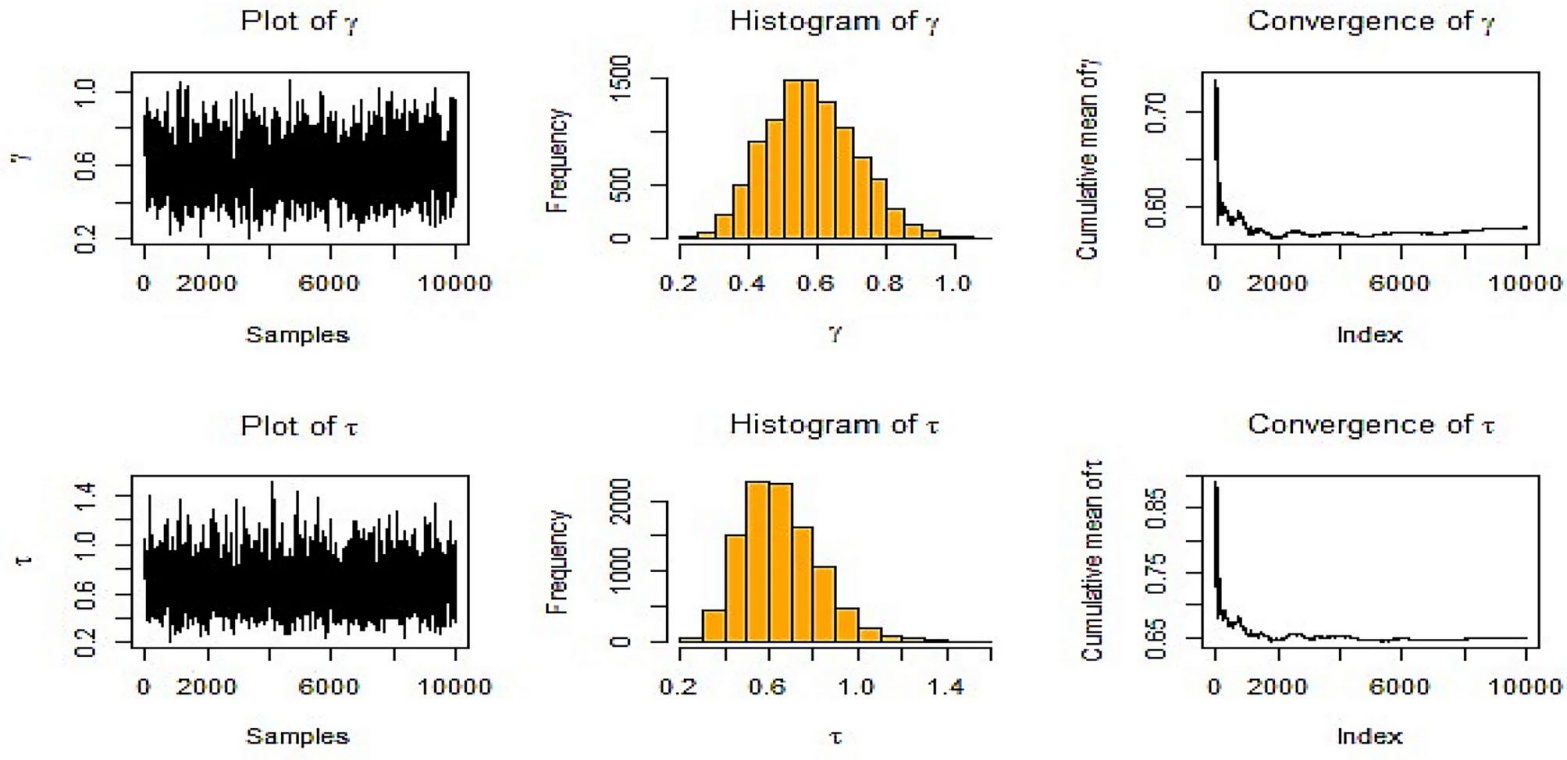
BE at true value $\gamma = 0.5$ and $\tau = 1.2$ at *m* = 3, *n* = 10 for NIFP in MRSSU.
- In the case of IFPs, history plots for various estimates $\gamma ,$ and $\tau$ are shown in Fig. 5. Indicative of convergence, the plots of the chains for the parameters create a horizontal band with no discernible long-term upward or falling trends.

In this subsection, study results can be summarized in Figs. 2, 3, 4, 5, 6, 7, 8, and 9 and Tables 1, 2, 3, 4, 5, and 6 as follows:

- Fig. 2 shows history graphs for various estimates of $\gamma ,$ and $\tau$ in the instance of NIFP in RSS. The parameters $\gamma ,$ and $\tau$ chain plots resemble a horizontal band with short upward and downward trends, which are signs of convergence.
  **Fig. 2:**
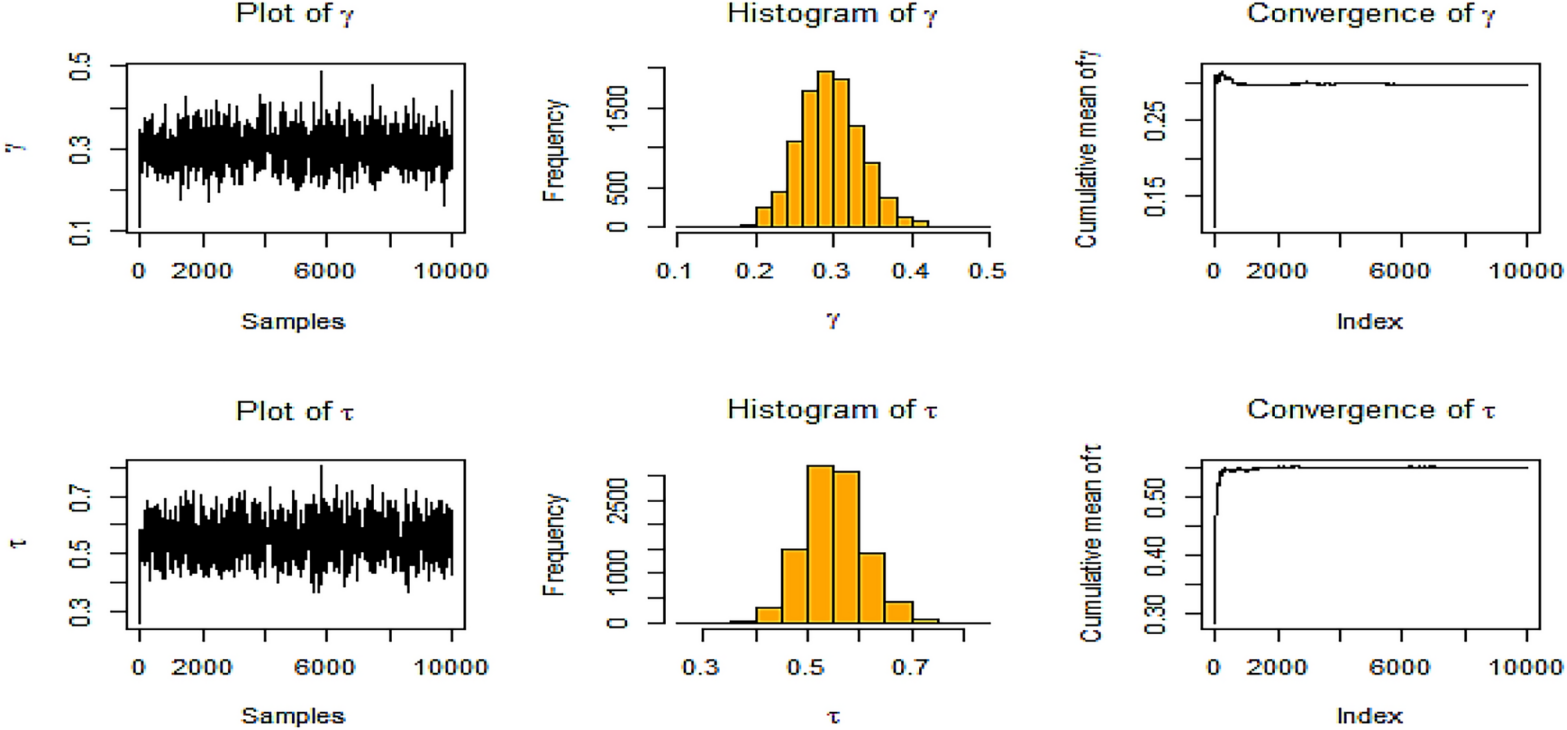
BE at true value $\gamma = 0.5$ and $\tau = 0.8$ at *m* = 3, *n* = 10 for NIFP in RSS*.*
- When IFP is used, Fig. 3 shows the history plot for many estimates for $\gamma ,$ and $\tau .$ The chain plots for the parameters $\gamma ,$ and $\tau$ appear to be a horizontal band without any discernible extended upward or downward trends, which are signs of convergence.
  **Fig. 3:**
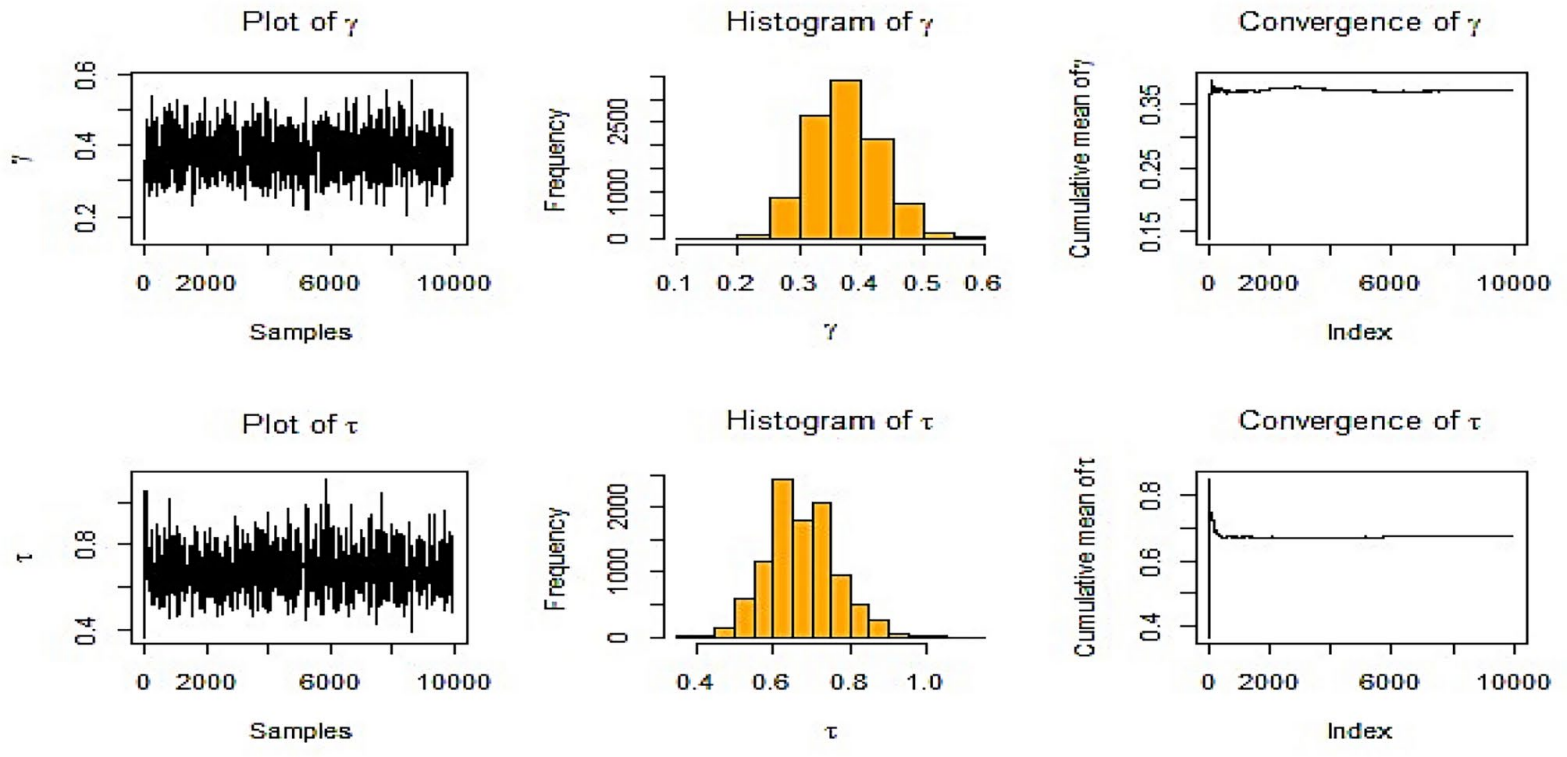
BE at true value $\gamma = 0.5$ and $\tau = 0.8$ at *m* = 3, *n* = 10 for IFP in RSS.
- Fig. 4 illustrates a history graph for several estimates of $\gamma ,$ and $\tau$ in the contexts of NIFP in MRSSU. The plots of the chains for the parameters $\gamma ,$ and $\tau$ show no broad upward or falling trends, but rather resemble a horizontal band, which is indicative of convergence.

**Fig. 5:**
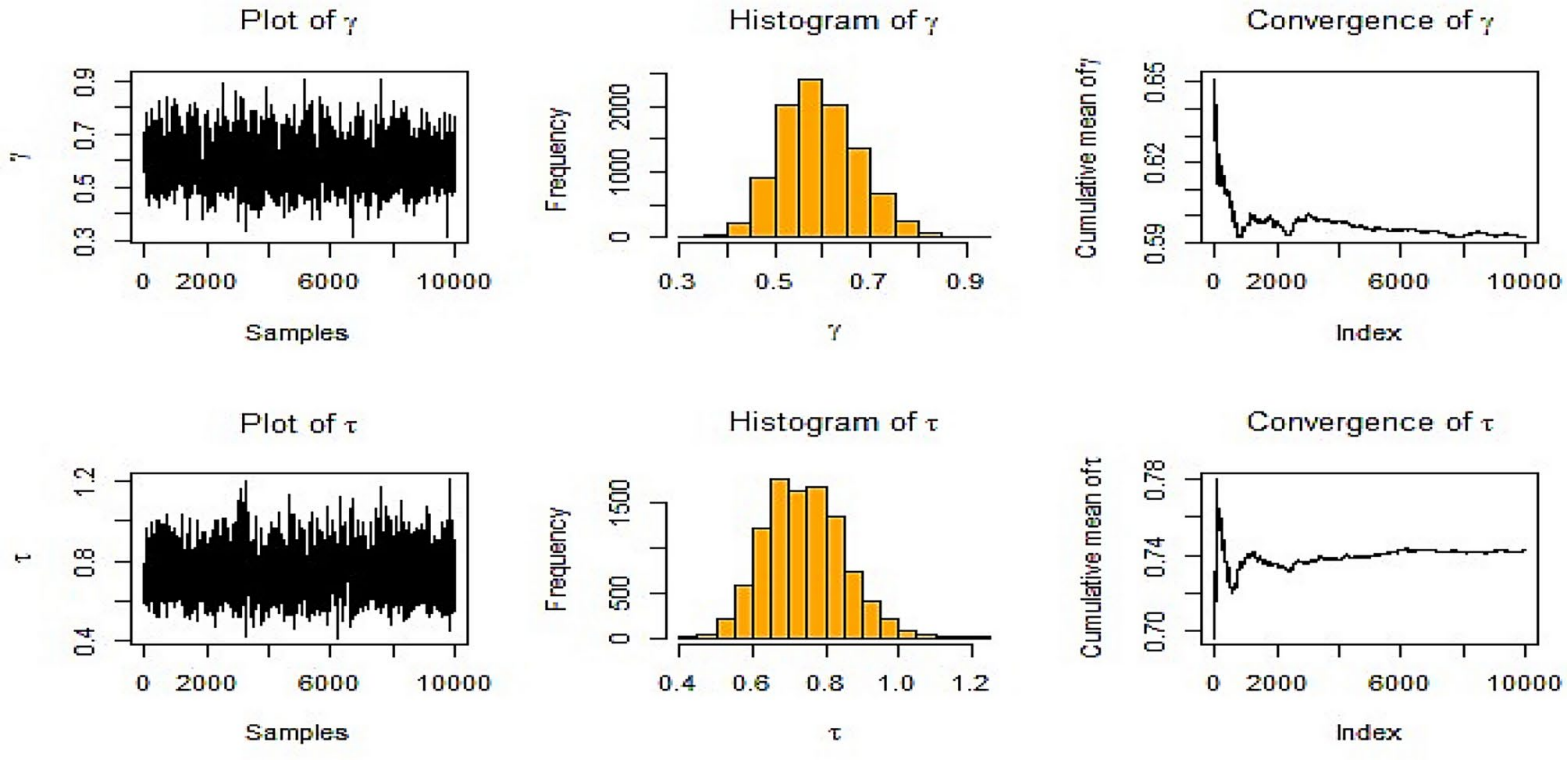
BE at true value $\gamma = 0.5$ and $\tau = 1.2$ at *m* = 3, *n* = 10 for IFP in MRSSU.

1. As sample size $n^\circ$ grows, the RMSE constantly goes down, showing that the estimates are all consistent.
2. As the sample size $n^\circ$ increases, the estimates’ precision increases, making them asymptotically unbiased.
3. The recommended estimates of $\gamma ,$ and $\tau$ generally approximate their genuine values as the sample size $n^\circ$ rises for fixed values of $\gamma ,$ and $\tau$.
4. The left panel of Fig. 6 shows that under INF prior, the RMSE of $\tilde{\gamma }_{SELoF} ,$ and $\tilde{\tau }_{SELoF}$ obtains the least values. Figure 6 right panel, however, demonstrates that in the case of NINF prior, the RMSE of $\tilde{\gamma }_{MELoF} ,$ and $\tilde{\tau }_{MELoF}$ receives the biggest value.

**Fig. 6:**
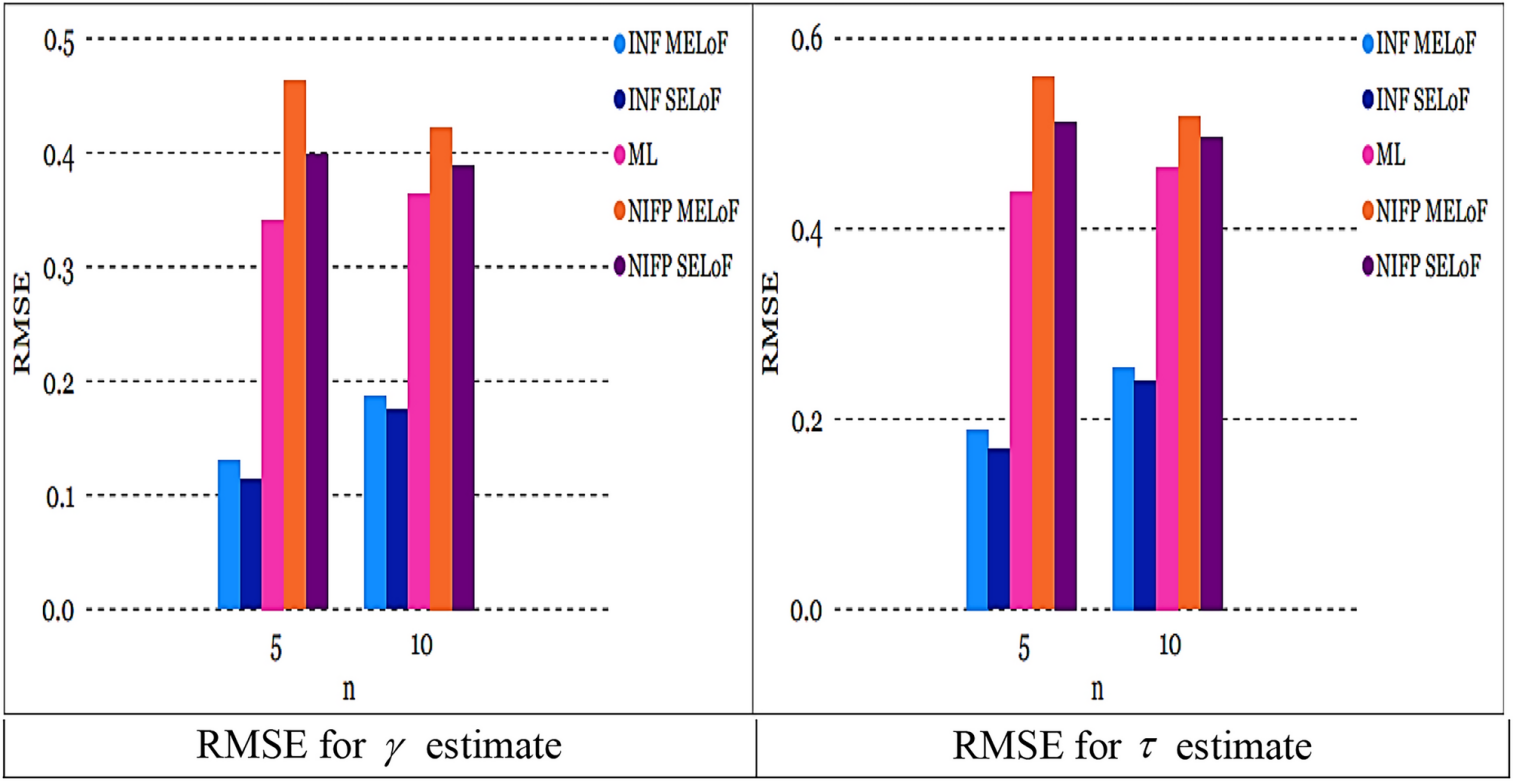
RMSE for all estimates in RSS at true value $\gamma = 0.5$ and $\tau = 0.8$.
5. The RMSE of $$\overset{\lower0.5em\hbox{$\smash{\scriptscriptstyle\frown}$}}{\gamma }_{SELoF} ,$$ and $$\overset{\lower0.5em\hbox{$\smash{\scriptscriptstyle\frown}$}}{\tau }_{MELoF}$$ takes the smallest value under INF, as can be shown from the left panel of Fig. 7.
6. However, as can be seen in the right panel of Fig. 7, under NIFP, the RMSE of $$\overset{\lower0.5em\hbox{$\smash{\scriptscriptstyle\frown}$}}{\gamma }_{SELoF}$$ receives the biggest value, while in the case of MLE, the RMSE of $$\hat{\tau }_{2}$$ receives the largest value.
7. The plots in Figs. 6 and 7 demonstrate that the RMSE of all estimates derived from MLE and BE decreases as the cycle number *n* grows.

**Fig. 7:**
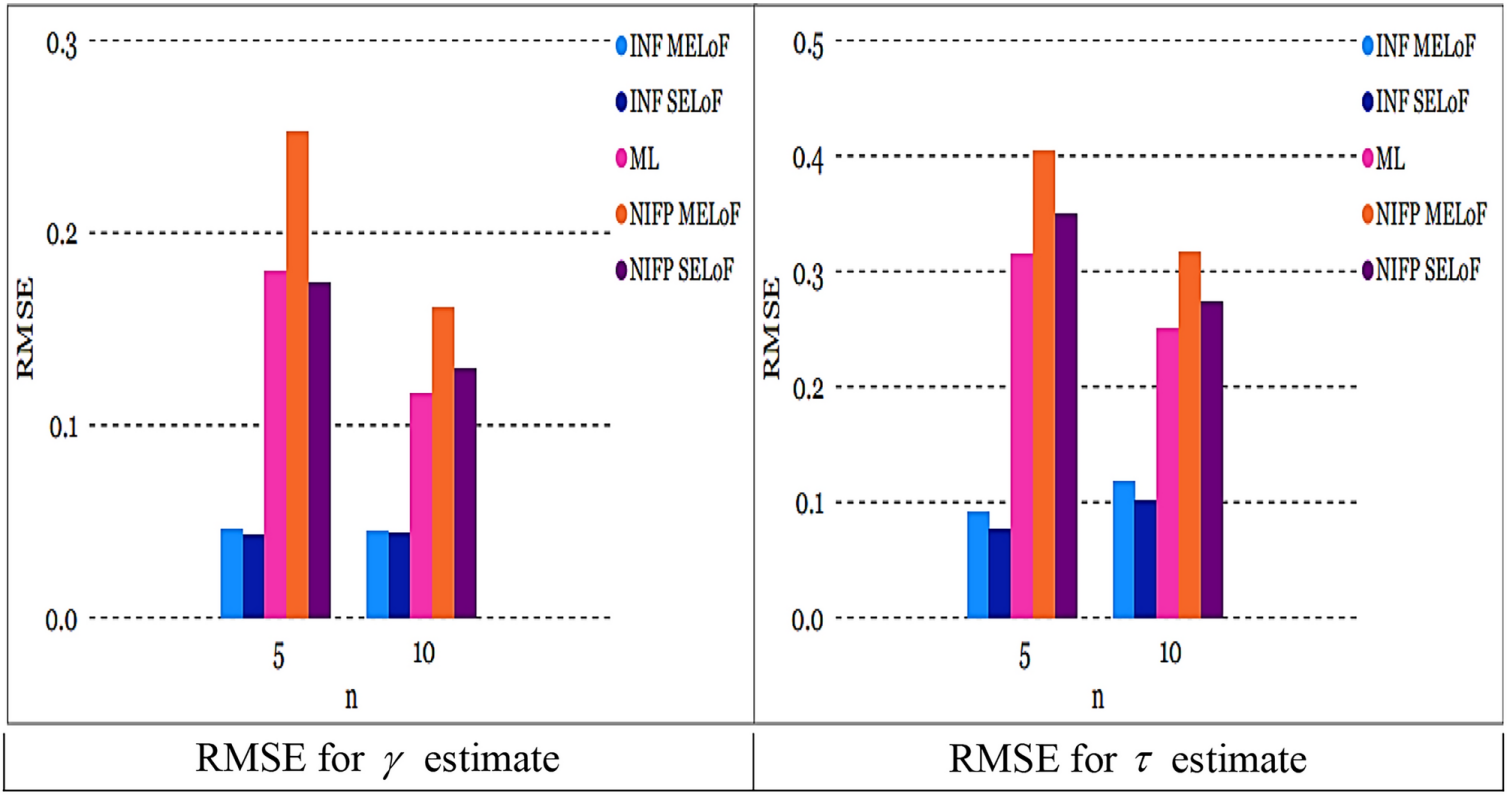
RMSE for all estimates in MRSSU at true value $\gamma = 0.5$ and $\tau = 0.8$
8. All estimates using MRSSU have a lower RMSE than estimates using RSS over a range of sample sizes; see Figs. 8 and 9.
9. In the majority of cases, all estimates derived from ML and Bayesian techniques based on MRSSU are preferred over the corresponding estimates derived from RSS in accordance with their RMSE values, see Tables 1, 2, 3, 4, 5, and 6.

**Fig. 8:**
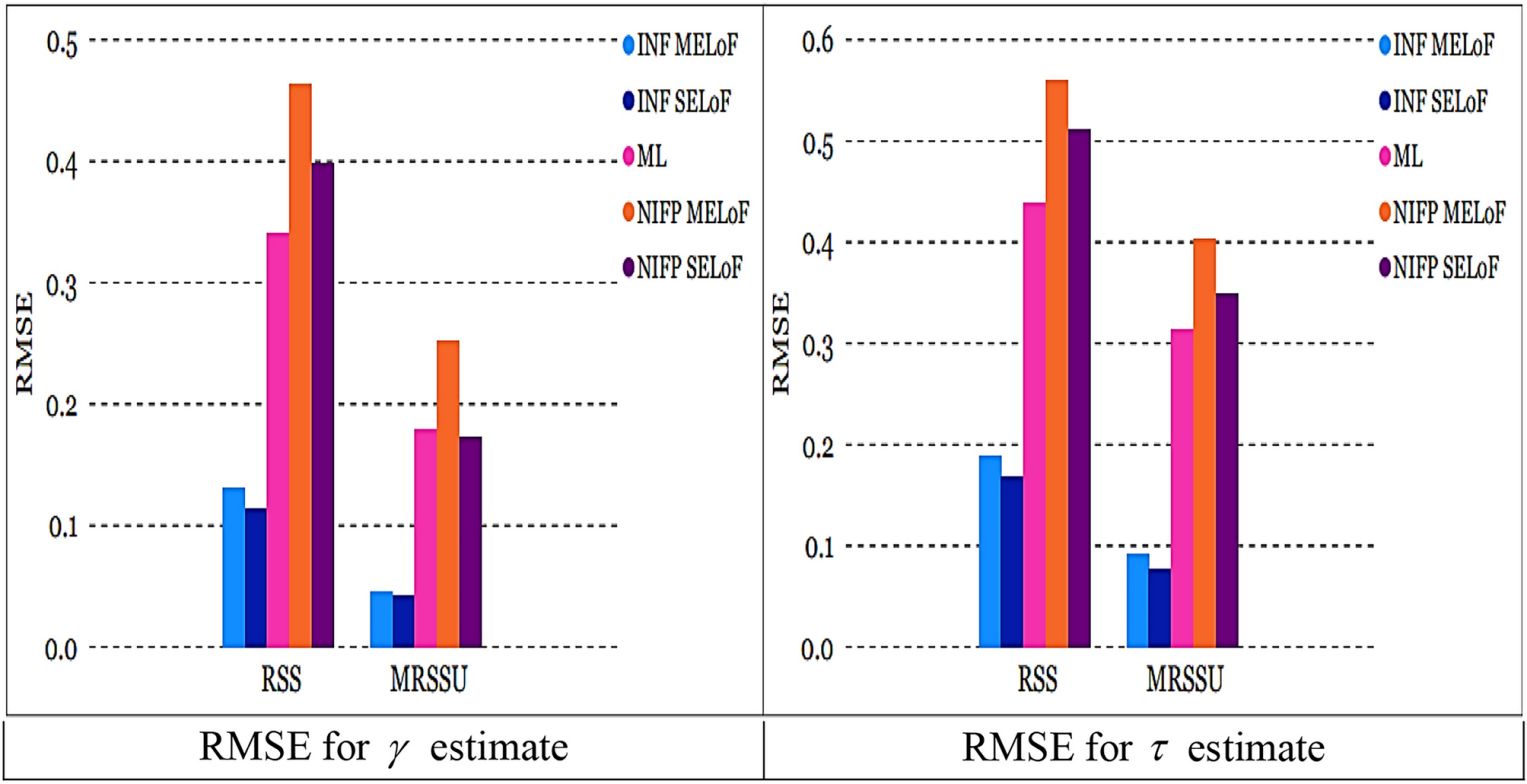
RMSE for all estimates at true value $\gamma = 0.5$ and $\tau = 0.8$ for *m* = 3*, n* = 5.

**Fig. 9:**
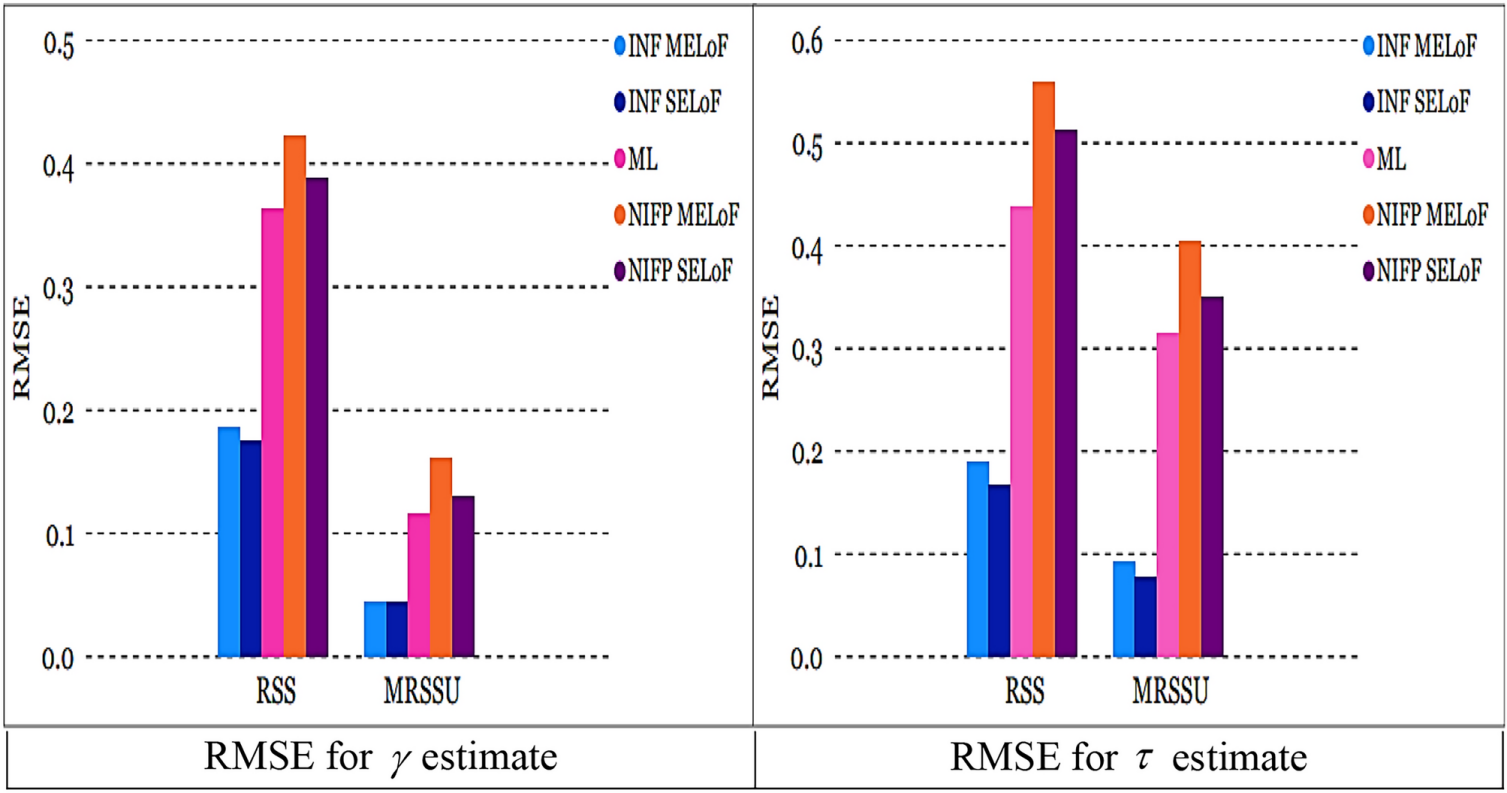
RMSE for all estimates at true value $\gamma = 0.5$ and $\tau = 0.8$ for *m* = 3, *n* = 10.

**Table 1:** RAB and RMSE for different estimates of parameters at $\gamma = 0.5$ and $\tau = 0.8$ in RSS.

*m*	*n*		MLE		NIFP				IFP			
					SELoF		MELoF		SELoF		MELoF	
			RMSE	RAB	RMSE	RAB	RMSE	RAB	RMSE	RAB	RMSE	RAB
2	5	$\gamma$	0.252	0.451	0.382	0.743	0.478	0.953	0.055	0.076	0.071	0.122
		$\tau$	0.331	0.369	0.600	0.595	0.606	0.745	0.083	0.069	0.105	0.108
	10	$\gamma$	0.288	0.559	0.339	0.668	0.408	0.809	0.094	0.170	0.110	0.205
		$\tau$	0.371	0.450	0.434	0.534	0.481	0.595	0.139	0.156	0.160	0.186
3	5	$\gamma$	0.341	0.674	0.398	0.792	0.463	0.924	0.114	0.214	0.131	0.250
		$\tau$	0.438	0.541	0.512	0.636	0.560	0.696	0.168	0.197	0.189	0.226
	10	$\gamma$	0.364	0.724	0.389	0.776	0.423	0.843	0.175	0.343	0.187	0.368
		$\tau$	0.465	0.578	0.497	0.619	0.518	0.646	0.241	0.295	0.254	0.313
4	5	$\gamma$	0.386	0.769	0.419	0.836	0.459	0.917	0.173	0.339	0.187	0.367
		$\tau$	0.491	0.611	0.536	0.668	0.563	0.702	0.243	0.296	0.259	0.318
	10	$\gamma$	0.405	0.809	0.420	0.839	0.439	0.878	0.246	0.490	0.255	0.508
		$\tau$	0.515	0.643	0.535	0.668	0.547	0.683	0.331	0.411	0.340	0.423
5	5	$\gamma$	0.439	0.877	0.458	0.916	0.479	0.958	0.241	0.479	0.251	0.500
		$\tau$	0.562	0.702	0.594	0.742	0.610	0.761	0.335	0.415	0.345	0.429
	10	$\gamma$	0.441	0.881	0.450	0.899	0.461	0.923	0.315	0.629	0.322	0.643
		$\tau$	0.564	0.704	0.578	0.723	0.586	0.732	0.416	0.519	0.422	0.526

**Table 2:** RAB and RMSE for different estimates of parameters at $\gamma = 0.5$ and $\tau = 0.8$ in MRSSU.

*m*	*n*		MLE		NIFP				IFP			
					SELoF		MELoF		SELoF		MELoF	
			RMSE	RAB	RMSE	RAB	RMSE	RAB	RMSE	RAB	RMSE	RAB
2	5	$\gamma$	0.303	0.215	0.243	0.232	0.377	0.676	0.041	0.022	0.040	0.025
		$\tau$	0.921	0.313	0.501	0.180	0.502	0.546	0.064	0.008	0.075	0.054
	10	$\gamma$	0.164	0.056	0.155	0.125	0.207	0.325	0.044	0.021	0.042	0.019
		$\tau$	0.316	0.008	0.286	0.163	0.323	0.327	0.072	0.025	0.083	0.062
3	5	$\gamma$	0.180	0.052	0.174	0.174	0.253	0.423	0.047	0.020	0.046	0.024
		$\tau$	0.415	0.017	0.350	0.221	0.404	0.447	0.077	0.033	0.092	0.076
	10	$\gamma$	0.117	0.049	0.130	0.149	0.161	0.252	0.044	0.006	0.045	0.026
		$\tau$	0.251	0.148	0.274	0.243	0.317	0.343	0.102	0.078	0.119	0.113
4	5	$\gamma$	0.143	0.035	0.156	0.184	0.206	0.337	0.046	0.021	0.044	0.013
		$\tau$	0.343	0.122	0.343	0.277	0.395	0.434	0.095	0.061	0.111	0.099
	10	$\gamma$	0.105	0.107	0.122	0.175	0.147	0.241	0.041	0.007	0.043	0.032
		$\tau$	0.271	0.244	0.298	0.308	0.335	0.378	0.129	0.121	0.145	0.150
5	5	$\gamma$	0.126	0.104	0.149	0.213	0.189	0.319	0.044	0.005	0.045	0.025
		$\tau$	0.331	0.252	0.359	0.358	0.410	0.471	0.121	0.107	0.140	0.142
	10	$\gamma$	0.108	0.153	0.126	0.205	0.145	0.252	0.043	0.026	0.046	0.047
		$\tau$	0.302	0.332	0.330	0.377	0.363	0.428	0.162	0.173	0.178	0.198

**Table 3:** RAB and RMSE for different estimates of parameters at $\gamma = 0.5$ and $\tau = 1.2$ in RSS.

*m*	*n*		MLE		NIFP				IFP			
					SELoF		MELoF		SELoF		MELoF	
			RMSE	RAB	RMSE	RAB	RMSE	RAB	RMSE	RAB	RMSE	RAB
2	5	$\gamma$	0.235	0.421	0.345	0.670	0.448	0.890	0.046	0.062	0.058	0.097
		$\tau$	0.532	0.398	0.741	0.600	0.889	0.733	0.140	0.082	0.179	0.126
	10	$\gamma$	0.269	0.522	0.317	0.623	0.374	0.741	0.081	0.146	0.093	0.173
		$\tau$	0.597	0.485	0.686	0.564	0.758	0.626	0.225	0.169	0.259	0.202
3	5	$\gamma$	0.321	0.634	0.374	0.743	0.434	0.864	0.097	0.181	0.109	0.208
		$\tau$	0.699	0.577	0.797	0.660	0.864	0.717	0.260	0.204	0.294	0.235
	10	$\gamma$	0.343	0.683	0.367	0.732	0.396	0.790	0.156	0.307	0.166	0.327
		$\tau$	0.739	0.614	0.783	0.651	0.814	0.677	0.393	0.322	0.415	0.341
4	5	$\gamma$	0.366	0.728	0.398	0.793	0.435	0.868	0.154	0.301	0.164	0.323
		$\tau$	0.778	0.646	0.838	0.696	0.876	0.729	0.394	0.322	0.420	0.345
	10	$\gamma$	0.385	0.769	0.400	0.799	0.417	0.833	0.224	0.446	0.232	0.461
		$\tau$	0.814	0.678	0.841	0.700	0.858	0.715	0.535	0.444	0.550	0.456
5	5	$\gamma$	0.421	0.841	0.441	0.881	0.463	0.924	0.219	0.434	0.227	0.451
		$\tau$	0.881	0.734	0.921	0.767	0.942	0.785	0.538	0.445	0.555	0.460
	10	$\gamma$	0.423	0.845	0.432	0.864	0.443	0.886	0.292	0.583	0.298	0.594
		$\tau$	0.884	0.737	0.903	0.752	0.913	0.761	0.665	0.554	0.675	0.561

**Table 4:** RAB and RMSE for different estimates of parameters at $\gamma = 0.5$ and $\tau = 1.2$ in MRSSU.

*m*	*n*		MLE		NIFP				IFP			
					SELoF		MELoF		SELoF		MELoF	
			RMSE	RAB	RMSE	RAB	RMSE	RAB	RMSE	RAB	RMSE	RAB
2	5	$\gamma$	0.264	0.181	0.222	0.209	0.330	0.552	0.473	0.058	0.088	0.010
		$\tau$	1.756	0.404	1.152	0.130	0.833	0.540	0.110	0.009	0.156	0.070
	10	$\gamma$	0.146	0.043	0.142	0.124	0.185	0.281	0.039	0.020	0.037	0.011
		$\tau$	0.535	0.023	0.452	0.168	0.512	0.353	0.114	0.026	0.133	0.068
3	5	$\gamma$	0.162	0.040	0.164	0.174	0.227	0.367	0.041	0.020	0.039	0.013
		$\tau$	0.718	0.041	0.558	0.229	0.642	0.475	0.129	0.038	0.157	0.089
	10	$\gamma$	0.108	0.051	0.122	0.147	0.149	0.231	0.039	0.007	0.039	0.018
		$\tau$	0.408	0.150	0.437	0.255	0.507	0.368	0.162	0.082	0.191	0.121
4	5	$\gamma$	0.132	0.039	0.148	0.179	0.190	0.303	0.040	0.017	0.039	0.010
		$\tau$	0.573	0.115	0.567	0.281	0.630	0.459	0.151	0.067	0.179	0.109
	10	$\gamma$	0.099	0.105	0.117	0.170	0.138	0.225	0.037	0.006	0.038	0.026
		$\tau$	0.430	0.254	0.473	0.323	0.533	0.401	0.203	0.127	0.231	0.159
5	5	$\gamma$	0.119	0.103	0.143	0.208	0.177	0.298	0.039	0.009	0.038	0.015
		$\tau$	0.535	0.257	0.567	0.373	0.651	0.499	0.201	0.120	0.234	0.161
	10	$\gamma$	0.103	0.149	0.121	0.199	0.137	0.239	0.039	0.024	0.042	0.041
		$\tau$	0.476	0.348	0.521	0.397	0.574	0.453	0.256	0.183	0.284	0.212

**Table 5:** RAB and RMSE for different estimates s of parameters at $\gamma = 1.5$ and $\tau = 1.5$ in RSS.

*m*	*n*		MLE		NIFP				IFP			
					SELoF		MELoF		SELoF		MELoF	
			RMSE	RAB	RMSE	RAB	RMSE	RAB	RMSE	RAB	RMSE	RAB
2	5	$\gamma$	0.681	0.406	1.000	0.646	1.364	0.903	0.135	0.061	0.173	0.096
		$\tau$	0.690	0.415	0.947	0.614	1.148	0.759	0.193	0.091	0.248	0.142
	10	$\gamma$	0.780	0.504	0.916	0.600	1.088	0.718	0.226	0.135	0.259	0.160
		$\tau$	0.774	0.504	0.882	0.580	0.974	0.645	0.296	0.179	0.341	0.213
3	5	$\gamma$	0.934	0.614	1.086	0.719	1.291	0.857	0.263	0.163	0.297	0.188
		$\tau$	0.903	0.597	1.018	0.676	1.106	0.735	0.344	0.217	0.390	0.188
	10	$\gamma$	0.999	0.663	1.069	0.710	1.154	0.767	0.443	0.289	0.470	0.308
		$\tau$	0.954	0.634	1.006	0.669	1.046	0.696	0.515	0.338	0.544	0.358
4	5	$\gamma$	1.066	0.708	1.159	0.770	1.279	0.851	0.435	0.284	0.465	0.305
		$\tau$	1.002	0.666	1.071	0.713	1.120	0.746	0.516	0.338	0.551	0.361
	10	$\gamma$	1.126	0.749	1.168	0.778	1.221	0.813	0.643	0.426	0.663	0.440
		$\tau$	1.046	0.697	1.078	0.718	1.100	0.733	0.697	0.462	0.716	0.475
5	5	$\gamma$	1.236	0.823	1.294	0.862	1.371	0.913	0.625	0.413	0.648	0.429
		$\tau$	1.128	0.752	1.173	0.782	1.201	0.800	0.699	0.463	0.721	0.478
	10	$\gamma$	1.241	0.827	1.269	0.846	1.304	0.869	0.844	0.561	0.860	0.572
		$\tau$	1.132	0.754	1.153	0.768	1.166	0.777	0.862	0.574	0.873	0.582

**Table 6:** RAB and RMSE for different estimates of parameters at $\gamma = 1.5$ and $\tau = 1.5$ in MRSSU.

*m*	*n*		MLE		NIFP				IFP			
					SELoF		MELoF		SELoF		MELoF	
			RMSE	RAB	RMSE	RAB	RMSE	RAB	RMSE	RAB	RMSE	RAB
2	5	$\gamma$	0.747	0.168	0.616	0.208	1.004	0.586	0.113	0.016	0.111	0.021
		$\tau$	2.544	0.469	1.276	0.135	1.022	0.591	0.149	0.004	0.173	0.066
	10	$\gamma$	0.417	0.038	0.403	0.120	0.525	0.268	0.108	0.019	0.102	0.009
		$\tau$	0.721	0.033	0.605	0.164	0.663	0.368	0.150	0.026	0.175	0.071
3	5	$\gamma$	0.465	0.035	0.461	0.169	0.649	0.360	0.111	0.019	0.105	0.010
		$\tau$	0.980	0.058	0.734	0.225	0.825	0.499	0.169	0.038	0.205	0.092
	10	$\gamma$	0.312	0.051	0.352	0.142	0.427	0.222	0.108	0.006	0.109	0.016
		$\tau$	0.534	0.150	0.563	0.258	0.652	0.381	0.211	0.084	0.248	0.126
4	5	$\gamma$	0.383	0.040	0.424	0.177	0.544	0.296	0.110	0.017	0.105	0.007
		$\tau$	0.766	0.109	0.706	0.290	0.805	0.483	0.198	0.069	0.234	0.114
	10	$\gamma$	0.288	0.104	0.340	0.167	0.397	0.219	0.101	0.006	0.105	0.024
		$\tau$	0.556	0.260	0.608	0.331	0.687	0.416	0.264	0.131	0.300	0.166
5	5	$\gamma$	0.348	0.102	0.416	0.205	0.510	0.289	0.108	0.009	0.107	0.012
		$\tau$	0.701	0.259	0.728	0.381	0.835	0.517	0.259	0.124	0.304	0.167
	10	$\gamma$	0.302	0.147	0.352	0.195	0.399	0.233	0.107	0.023	0.116	0.039
		$\tau$	0.612	0.357	0.667	0.407	0.738	0.467	0.331	0.189	0.367	0.219

## Application for real data

This section considers and provides a detailed explanation of an actual data set to illustrate the applicability of the proposed estimators. The data set was reported by Nagy et al.^28^. The data set was gathered in 1998 based on the 64 consecutive eruptions of Kiama Blowhole. The Kiama Blowhole, located around 120 km south of Sydney, is a well-liked tourist attraction. Water is forced through an opening in a cliff by the surge of the ocean. Then, out of nowhere, the water bursts through an opening, frequently soaking everyone in its path.1340 h of eruption data have been collected since 12 July 1998. These are the specifications for the data set:

**Table:** 

51	83	87	60	28	95	8	27	15	10
18	16	29	54	91	8	17	55	10	35
47	77	36	17	21	36	18	40	10	7
34	27	28	56	8	25	68	146	89	18
73	69	9	37	10	82	29	8	60	61
61	18	169	25	8	26	11	83	11	42
17	14	9	12						

**Fig. 10:**
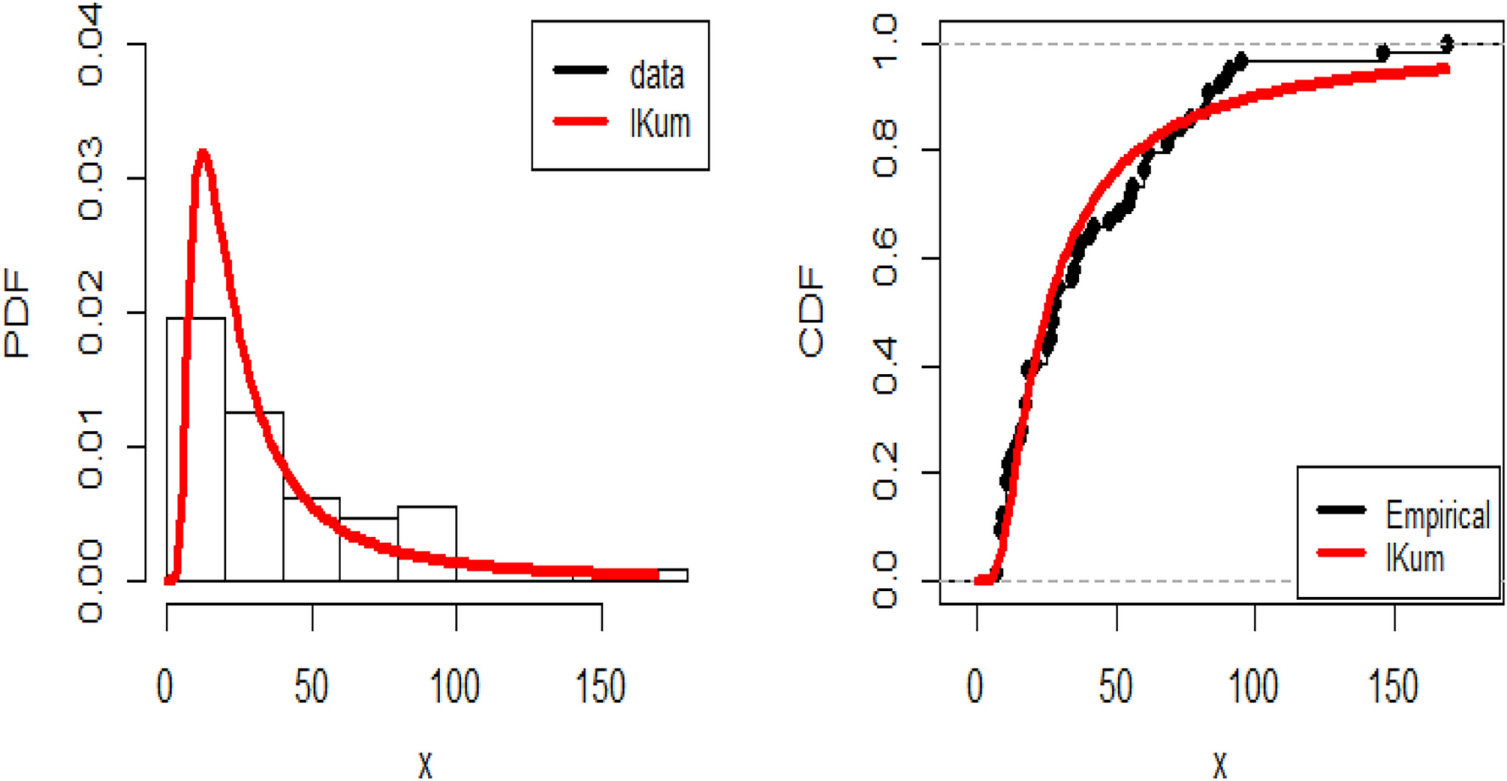
Estimated PDF and CDF Plots for IKmD.

The Kolmogorov–Smirnov (K–S) test is used to determine whether a fitted model exists for the data set, and the maximum likelihood approach is then utilized to compute the estimates. The K–S distance is 0.097 with a p-value of 0.586. It follows that the IKmD is an appropriate model for fitting this data. Figure 10 shows the estimated PDF and empirical CDF for the data. Based on this graph, it appears that the IKmD model fits the data well.

The data contain outliers, and we treated them by replacing outliers with the median of the data. The MRSSU and RSS sampling approaches are used to examine actual data sets based on the aforementioned theoretical findings. Tables 7 and 8 discuss the MLE and BE along with their standard errors (StEs) based on RSS and MRSSU, respectively, from the IKmD for different set sizes *m* and number of cycles five and ten. We employed NIFP to calculate the BE because we have no knowledge of the priors; thus, we chose. The MRSSU and RSS observations are produced using the R-package.

**Table 7:** MLE, BE and StEs for the data set using RSS.

*m*	*n*		MLE		SELoF		MELoF	
			Estimate	StEs	Estimate	StEs	Estimate	StEs
2	5	$\gamma$	1.863	0.354	1.420	0.164	1.376	0.178
		$\tau$	240.027	245.427	66.729	24.218	44.654	22.655
	10	$\gamma$	1.539	0.221	1.681	0.164	1.649	0.162
		$\tau$	89.178	58.664	153.048	67.675	107.805	45.683
3	5	$\gamma$	1.706	0.245	1.863	0.174	1.832	0.168
		$\tau$	80.139	51.911	135.657	60.890	101.274	37.642
	10	$\gamma$	1.610	0.161	1.495	0.132	1.470	0.137
		$\tau$	73.763	32.665	56.481	18.897	42.021	17.590
4	5	$\gamma$	1.580	0.172	1.709	0.146	1.684	0.144
		$\tau$	75.820	37.313	119.871	45.727	91.922	33.422

**Table 8:** MLE, BE and StEs for the data set using MRSSU.

*m*	*n*		MLE		SELoF		MELoF	
			Estimate	StEs	Estimate	StEs	Estimate	StEs
2	5	$\gamma$	1.208	0.302	0.832	0.250	0.679	0.229
		$\tau$	25.721	22.322	9.534	8.095	2.943	2.530
	10	$\gamma$	1.471	0.250	1.147	0.150	1.107	0.148
		$\tau$	57.079	40.699	22.763	9.565	17.161	6.482
3	5	$\gamma$	1.330	0.268	1.045	0.177	0.986	0.169
		$\tau$	27.594	21.292	12.557	6.339	8.071	3.844
	10	$\gamma$	1.252	0.179	1.247	0.122	1.223	0.123
		$\tau$	23.115	12.210	24.055	8.165	19.193	6.593
4	5	$\gamma$	1.829	0.257	1.591	0.157	1.560	0.156
		$\tau$	339.952	302.543	159.607	82.343	99.681	49.020

**Fig. 11:**
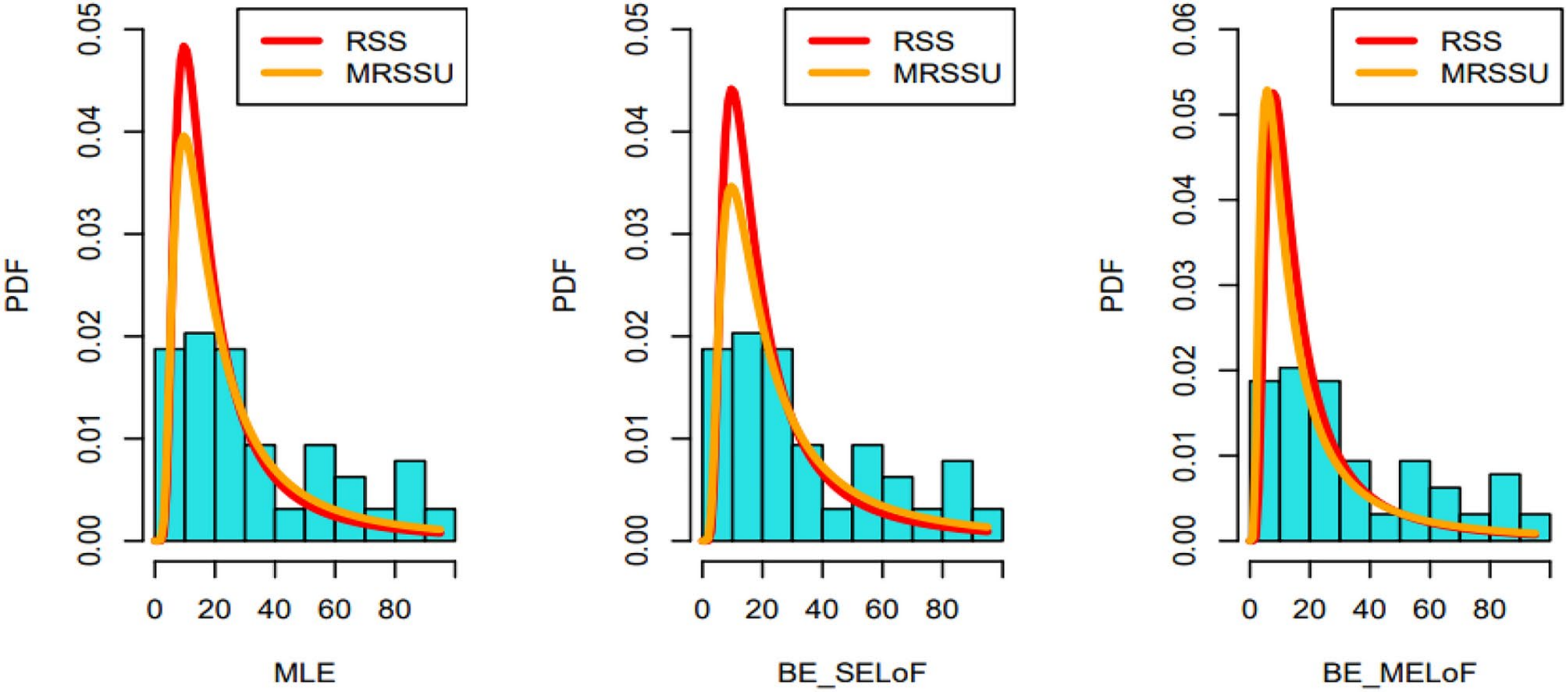
Plots of the estimated PDFs under RSS and MRSSU designs for the dataset.

**Fig. 12:**
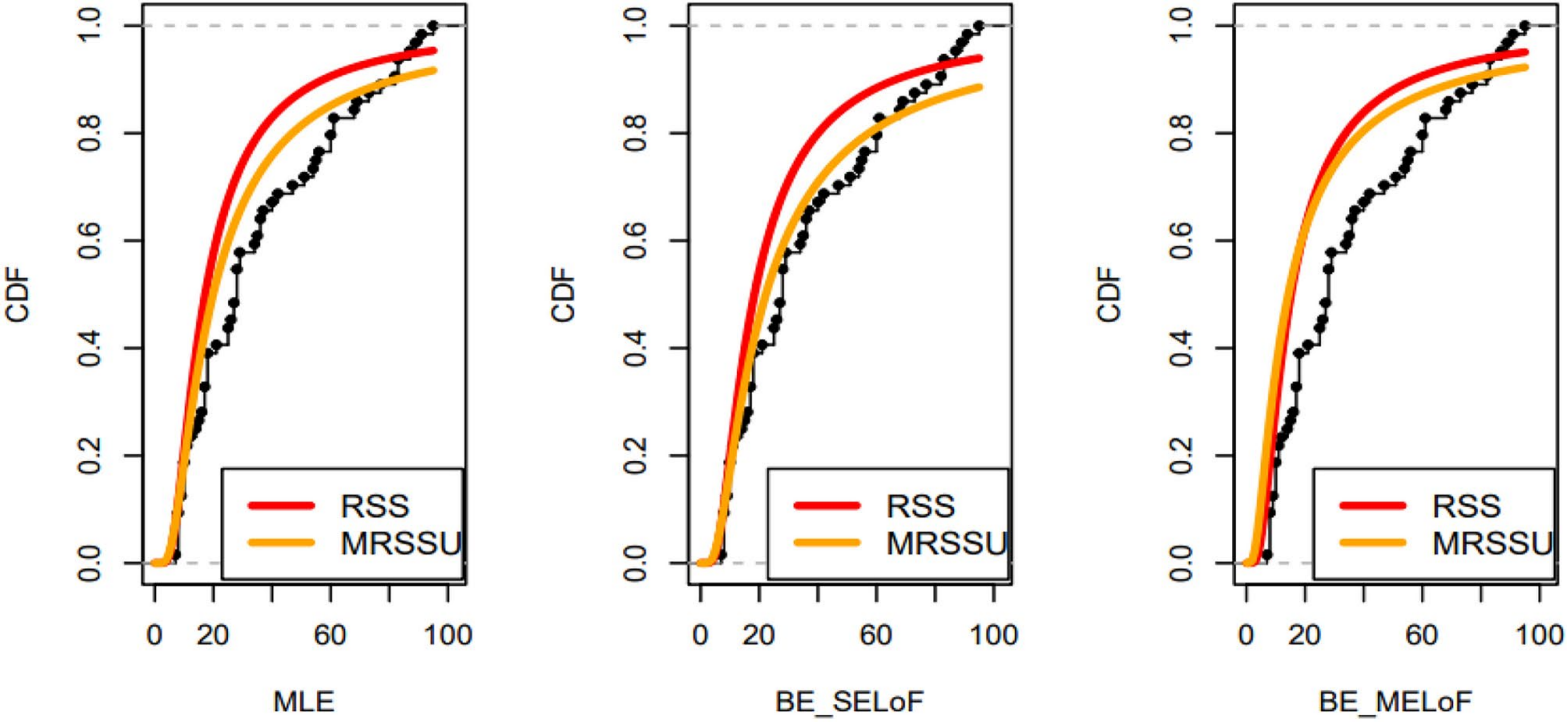
Plots of the estimated CDFs under RSS and MRSSU designs for the dataset.

For this dataset and each estimating method, the RSS and MRSSU design is taken into account, with the estimates being computed using a three-set size and two number of cycles. Figures 11 and 12 show the corresponding fittings for RSS and MRSSU.

## Conclusions

It has been demonstrated that RSS is a more effective substitute for SRS, achieving the same level of precision with a smaller size of sample than required for SRS. Furthermore, figuring out each set’s maximum is easy. An extremely helpful variation of RSS is MRSSU. It allows a larger set size to be used without creating too many ranking errors. The MRSSU is frequently employed in life testing and reliability studies. The primary objective of this article is to estimate the parameters of the IKmD using the RSS and MRSSU methods. Bayesian in the case of non-informative and informative priors and ML estimation methods are used. Bayes estimates are calculated under minimum expected and squared error loss functions. The numerical simulation results demonstrate that Bayes estimate performs better than MLE relative to their RMSE’s and RAB’s. In the case of an INF prior, the BEs under SELoF are superior to the similar under MELoF with respect to the lowest values of RMSE and RAB. With the same sample sizes, the suggested MRSSU estimators outperform their RSS counterparts in terms of the least RMSE and RAB for all results shown in the tables. Since the estimates accuracy increases with sample size, then they are asymptotically unbiased. The MRSSU design outperformed the RSS design in numerical simulation and real dataset findings.

## Data Availability

The data sets used and analyzed during the current study are available to readers as in this article.

## References

[1] McIntyre, G. A. A method for unbiased selective sampling, using ranked sets. *Aust. J. Agric. Res.* **3**, 385–390 (1952).

[2] Taylan, P., Weber, G. W. & Beck, A. New approaches to regression by generalized additive models and continuous optimization for modern applications in finance, science and technology. *Optimization* **56**(5–6), 675–698 (2007).

[3] Husby, C. E., Wolfe, D. A. & Stasny, E. A. An application of ranked set sampling for mean and median estimation using USDA crop production data. *Qual. Control Appl. Stat.* **51**(2), 159–160 (2006).

[4] Mode, N. A., Conquest, L. L. & Marker, D. A. Ranked set sampling for ecological research: Accounting for the total costs of sampling. *Environ. Off. J. Int. Environ. Soc.* **10**(2), 179–194 (1999).

[5] Ozturk, O., Bilgin, O. C. & Wolfe, D. A. Estimation of population mean and variance in flock management: A ranked set sampling approach in a finite population setting. *J. Stat. Comput. Simul.* **75**(11), 905–919 (2005).

[6] Bocci, C., Petrucci, A. & Rocco, E. Ranked set sampling allocation models for multiple skewed variables: An application to agricultural data. *Environ. Ecol. Stat.* **17**, 333–345 (2010).

[7] Halls, L. K. & Dell, T. R. Trial of ranked-set sampling for forage yields. *For. Sci.* **12**(1), 22–26 (1966).

[8] Nadarajah, S. & Kotz, S. Reliability for some bivariate exponential distributions. *Math. Probl. Eng.* **1**, 1–14 (2006).

[9] Lawless, J. F. *Statistical Models and Methods for Lifetime Data* (Wiley, 2011).

[10] Takahasi, K. & Wakimoto, K. On unbiased estimates of the population mean based on the sample stratified by means of ordering. *Ann. Inst. Stat. Math.* **20**(1), 1–31 (1968).

[11] Dell, T. R. & Clutter, J. L. Ranked set sampling theory with order statistics background. *Biometrics* **28**, 545–555 (1972).

[12] Lam, K., Sinha, B. K. & Wu, Z. Estimation of parameters in two-parameter exponential distribution using ranked set sampling. *Ann. Inst. Stat. Math.* **46**, 723–736 (1994).

[13] Stokes, L. Parametric ranked set sampling. *Ann. Inst. Stat. Math.* **47**, 465–482 (1995).

[14] Biradar, B. S. & Santosha, C. D. Estimation of the mean of the exponential distribution using maximum ranked set sampling with unequal samples. *Open J. Stat.* **4**, 641–649 (2014).

[15] Jiang, H. & Gui, W. Bayesian inference for the parameters of Kumaraswamy distribution via ranked set sampling. *Symmetry* **13**(7), 1170. 10.3390/sym13071170 (2021).

[16] Eskandarzadeh, M., Tahmasebi, S. & Hosseini. E. H. Bayesian estimation of the Exponential distribution based on maximum ranked set sampling with unequal samples. In: Proc. *47th Annual Iranian Mathematics Conference Kharazmi University*, (2016).

[17] Eskandarzadeha, M., Crescenzob, A. D. & Tahmasebia, S. Measures of information for maximum ranked set sampling with unequal sample. *Commun. Stat. Theory Methods* **47**(19), 4692–4709. 10.1080/03610926.2018.1445857 (2018).

[18] El-Din, M. M., Kotb, M. S. & Newer, H. A. Inference for linear exponential distribution based on record ranked set sampling. *J. Stat. Appl. Probab.* **10**(2), 512–524 (2021).

[19] Salehi, M. & Ahmadi, J. Record ranked set sampling scheme. *Metron* **72**(3), 351–365. 10.1007/s40300-014-0038-z (2014).

[20] Hassan, A. S., Elgarhy, M., Chesneau, C. & Nagy, H. F. Bayesian analysis of multi-component stress-strength reliability using improved record values. *J. Auton. Intell.* **7**(4), 1–20. 10.32629/jai.v7i4.868 (2024).

[21] Sadek, A., Sultan, K. S. & Balakrishnan, N. Bayesian estimation based on ranked set sampling using asymmetric loss function. *Bull. Malays. Math. Sci. Soc.* **38**, 707–718 (2009).

[22] Helu, A., Abu-Salih, M. & Alkam, O. Bayes estimation of Weibull distribution parameters using ranked set sampling. *Commun. Stat. Theory Methods* **39**(14), 2533–2551 (2010).

[23] Hassan, A. S. Maximum likelihood and Bayes estimators of the unknown parameters for exponentiated exponential distribution using ranked set sampling. *Int. J. Eng. Res. Appl.* **3**(1), 720–725 (2013).

[24] Sadek, A. & Alharbi, F. Weibull-Bayesian analysis based on ranked set sampling. *Int. J. Adv. Stat. Probab.* **2**, 114–123 (2014).

[25] Biradar, B. S. & Shivanna, B. K. Weibull-Bayesian estimation based on maximum ranked set sampling with unequal samples. *Open J. Stat.* **6**, 1028–1036 (2016).

[26] Bantan, R., Hassan, A. S. & Elsehetry, M. Zubair Lomax distribution: Properties and estimation based on ranked set sampling. *CMC-Comput. Mater. Continua* **65**(3), 2169–2187 (2020).

[27] Almarashi, A. M. et al. A new estimation study of the stress-strength reliability for the Topp-Leone distribution using advanced sampling methods. *Sci. Programm.* **2021**(1), 2404997 (2021).

[28] Nagy, H. F., Al-Omari, A. I., Hassan, A. S. & Alomani, G. A. Improved estimation of the inverted Kumaraswamy distribution parameters based on ranked set sampling with an application to real data. *Mathematics* **10**(21), 4102 (2022).

[29] Hassan, A. S., Elshaarawy, R. S. & Nagy, H. F. Parameter estimation of exponentiated exponential distribution under selective ranked set sampling. *Stat. Trans. New Series* **23**(4), 37–58 (2022).

[30] Chaudhary, S. K. & Gupta, N. General weighted extropy of minimum and maximum ranked set sampling with unequal samples. *arXiv preprint arXiv,2305.01227* (2023).

[31] Wang, S., Chen, W., Chen, M. & Zhou, Y. Maximum likelihood estimation of the parameters of the inverse Gaussian distribution using maximum rank set sampling with unequal samples. *Math. Popul. Stud.* **30**(1), 1–21 (2023).

[32] Hassan, A. S., Abd-Elfattah, A. M. & Nagy, H. F. Modified goodness of fit tests for the Weibull distribution based on moving extreme ranked set sampling. *In: The 48th Annual Conference on Statistics, Computer Science and Operations Research, Faculty of Graduate Studies for Statistical Research, Cairo University* (2013).

[33] Özdemir, Y. A., Ebegil, M. & Gökpinar, F. A test statistic based on ranked set sampling for two normal means. *Commun. Stat. Simul. Comput.* **46**(10), 8077–8085 (2017).

[34] Al-Omari, A. I., Hassan, A. S., Alotaibi, N., Shrahili, M. & Nagy, H. F. Reliability estimation of inverse Lomax distribution using extreme ranked set sampling. *Adv. Math. Phys.* **1**, 4599872 (2021).

[35] Al-Omari, A. I., Benchiha, S. & Almanjahie, I. M. Efficient estimation of the generalized Quasi-Lindley distribution parameters under ranked set sampling and applications. *Math. Probl. Eng.*10.1155/2021/9982397 (2021).

[36] Al-Omari, A. I., Benchiha, S. & Almanjahie, I. M. Efficient estimation of two-parameter Xgamma distribution parameters using ranked set sampling design. *Mathematics* **10**(17), 3170 (2022).

[37] Hassan, A. S., Alsadat, N., Elgarhy, M., Chesneau, C. & Mohamed, R. E. Different classical estimation methods using ranked set sampling and data analysis for the inverse power Cauchy distribution. *J. Radiat. Res. Appl. Sci.* **16**(4), 100685 (2023).

[38] Alsadat, N., Hassan, A. S., Elgarhy, M., Chesneau, C. & Mohamed, R. E. An efficient stress–strength reliability estimate of the unit Gompertz distribution using ranked set sampling. *Symmetry* **15**(5), 1121 (2023).

[39] Alsadat, N., Hassan, A. S., Gemeay, A. M., Chesneau, C. & Elgarhy, M. Different estimation methods for the generalized unit half-logistic geometric distribution: Using ranked set sampling. *AIP Advances* **13**(8), 085230. 10.1063/5.0169140 (2023).

[40] Liu, J., Wang, L., Tripathi, Y. M. & Lio, Y. Inference of constant-stress model of Fréchet distribution under a maximum ranked set sampling with unequal samples. *Axioms* **13**(6), 394 (2024).

[41] Abd AL-Fattah, A. M., EL-Helbawy, A. A. & AL-Dayian, G. R. Inverted Kumaraswamy distribution: Properties and estimation. *Pakistan J. Stat.*, **33**, 37−61 (2017).

[42] Rashad, A., Yusuf, M. & Moheb, S. Approximate Bayes estimators of the inverted Kumaraswamy distribution parameters based on progressive Type-II censoring scheme. *J. Stat. Appl. Probab.* **8**(3), 189–199 (2019).

[43] Ravenzwaaij, D. V., Cassey, P. & Brown, S. D. A simple introduction to Markov Chain Monte-Carlo sampling. *Psychon. Bull. Rev.* **25**(1), 143–154 (2018). 26968853 10.3758/s13423-016-1015-8 PMC5862921

[44] Tummala, V. M. & Sathe, P. T. Minimum expected loss estimators of reliability and parameters of certain lifetime distributions. *IEEE Trans. Reliab.* **27**(4), 283–285 (1978).

[45] Tahmasebi, S., Hosseini, E. H. & Jafar, A. A. Bayesian estimation for Rayleigh distribution based on ranked set sampling. *New Trends Math. Sci.* **5**(4), 97–106 (2017).

[46] Dey, S., Singh, S., Tripathi, Y. M. & Asgharzadeh, A. Estimation and prediction for a progressively censored generalized inverted exponential distribution. *Stat. Methodol.* **32**, 185–202. 10.1080/08898480.2021.1996822 (2016).

[47] Dey, S. & Pradhan, B. Generalized inverted exponential distribution under hybrid censoring. *Stat. Methodol.* **18**, 101–114 (2014).

